# Structure elucidation and biological activities of perylenequinones from an *Alternaria* species

**DOI:** 10.1007/s12550-023-00495-1

**Published:** 2023-06-23

**Authors:** Anna Kiefer, Marcel Arnholdt, Viktoria Grimm, Leander Geske, Jonathan Groß, Nina Vierengel, Till Opatz, Gerhard Erkel

**Affiliations:** 1 Molecular Biotechnology & Systems Biology, RPTU, Paul-Ehrlich-Straße 23, D-67663 Kaiserslautern, Germany; 2 Department of Chemistry, Johannes Gutenberg-University, Duesbergweg 10-14, D-55128 Mainz, Germany

**Keywords:** Altertoxins, *Alternaria*, Perylenequinones, Oxidative stress

## Abstract

The KEAP1-Nrf2/ARE pathway is a pivotal cytoprotective regulator against oxidative stress which plays an important role in the development of many inflammatory diseases and cancer. Activation of the Nrf2 transcription factor by oxidative stress or electrophiles regulates antioxidant response element (ARE)-dependent transcription of antioxidative, detoxifying, and anti-inflammatory proteins. Therefore, modulators of the KEAP1-Nrf2/ARE pathway have received considerable interest as therapeutics to protect against diseases where oxidative stress constitutes the underlying pathophysiology. In a search for fungal secondary metabolites affecting the Nrf2/ARE-dependent expression of a luciferase reporter gene in BEAS-2B cells, three new perylenequinones, compounds **1**, **2**, and **3**, together with altertoxin-I (ATX-I), were isolated from fermentations of an *Alternaria* species. The structures of the compounds were elucidated by a combination of one- and two-dimensional NMR spectroscopy and mass spectrometry. Compound **1** and ATX-I exhibited strong cytotoxic effects with LC_50_-values of 3.8 µM and 6.43 µM, respectively, whereas compound **3** showed no cytotoxic effects up to 100 µM on BEAS-2B cells. ATX-I induced ARE-dependent luciferase expression approximately fivefold and compound **1** approximately 2.6-fold at a concentration of 3 µM in transiently transfected BEAS-2B cells. In addition, compound **1** and ATX-I exhibited strong oxidative effects, whereas compound **3** did not show significant oxidative properties. For compound **1** and ATX-I, a strong upregulation of heme oxygenase-1 could be observed on mRNA and protein level in treated BEAS-2B cells. Moreover, compound **3** significantly decreased *sod3* mRNA levels after induction of oxidative stress with benzoquinone.

**Supplementary Information:**

The online version contains supplementary material available at 10.1007/s12550-023-00495-1.

## Introduction

**Fig. 1:**
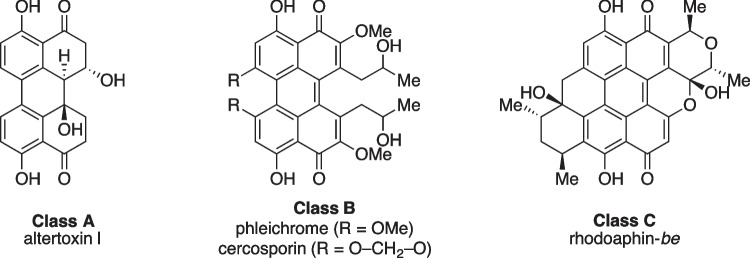
Classes A–C of perylenequinones: (A) without carbon substituents; (B) with carbon- containing substituents; (C) non-fungal perylenequinones

Perylenequinones are naturally occurring aromatic polyketides with an oxidized pentacyclic core, mostly found in various fungi but also in aphids, crinoids, and plants. Many of them have been isolated from *Alternaria* species, a highly diverse genus of molds (Khiralla et al. 2022). According to Weiss et al., natural perylenequinones can be categorized into three classes: Class A contains the simple perylenequinones without carbon substituents including altertoxin I (ATX-I), which are commonly produced by *Alternaria* species (Fig. 1). Class B consists of structures like cercosporin or phleichrome which all contain carbon substituents. Class C includes non-fungal pherylenequinones like rhodoaphin isolated from aphids (Weiss et al. 1987). *Alternaria* is a common genus of saprophytic or pathogenic fungi. They can be found on a variety of crop plants like cereals, fruits, and vegetables and are known to produce various mycotoxins like altertoxin I-III, tenuazonic acid, alternariol, and alternariol monomethyl ether (Lee et al. 2015). Due to the wide distribution of the fungus and the frequent infestation of food crops, alternaria toxins are considered as critical contaminants of food and feed (Arcella et al. 2016).

Whereas several successful total syntheses of members with an intact perylenequinone framework, like cercosporin (Morgan et al. 2009) or phleichrome (Morgan et al. 2010, 2009), have been achieved, only few attempts were made to synthesize altertoxins and related compounds ex vivo as they are not stable against elimination of water with formation of fully aromatic compounds. This and their tendency towards aggregation resulting in solubility problems make these structure a very challenging and a rarely tackled target for total synthesis (Geiseler et al. 2013; Pfaff et al. 2017).

For some perylenequinones and their precursors, especially those isolated from *Alternaria* sp., toxic effects have been described (Aichinger et al. 2021). For ATX-I, a mutagenic effect was confirmed with the Ames test in *Salmonella typhimurium* (Stack and Prival 1986). In addition, ATX-I inhibits topoisomerase II, which plays an essential role in maintaining the correct DNA topology (Jarolim et al. 2017a). Nevertheless, the exact mechanism of action of ATX-I genotoxicity has not yet been clarified.

Due to their photochemical activities, perylenequinones are discussed as possible drugs in photodynamic therapy (PDT) of cancer (Mulrooey et al. 2012). PDT is used to specifically induce cell death in cancer cells through photosensitizing compounds that generate reactive oxygen species upon irradiation (Agostinis et al. 2011; Fayter et al. 2010; Gao et al. 2010). Some perylenequinones like hypocrellin A and hypocrellin B showed beneficial properties for application in photodynamic therapy as they trigger cell death or decrease migration in cancer cell lines after irradiation (Qi et al. 2019; Jiang et al. 2014).

### Nrf2/ARE signaling pathway

Reactive oxygen species (ROS) are an unavoidable side effect of aerobic life. While elevated levels of ROS are toxic to cells and organisms, ROS also are an essential part of many cellular defense signaling pathways. Under physiological conditions, a constant regulation between formation and removal of ROS takes place, preventing the emergence of oxidative stress (Sies et al. 2017). Exposure to excessive oxidative stress drives inflammation, tumor growth, or even cell death (Sies and Jones 2020). One important coping mechanism for oxidative stress is the (nuclear factor erythroid-derived 2)-like 2/antioxidant response element (Nrf2/ARE) pathway. Under quiescent conditions, the transcription factor Nrf2 is located in the cytoplasm and is rapidly degraded. Proteolytic degradation is mediated by Kelch-like ECH-associated protein 1 (KEAP1), which catalyzes ubiquitination of Nrf2. In response to oxidative stress, degradation is inhibited, allowing Nrf2 to translocate into the nucleus, where it binds to ARE. Genes induced by Nrf2 encode proteins which stimulate NADPH synthesis, catalyze ROS degradation, accelerate toxin export, and inhibit cytokine-mediated inflammation (Hayes and McMahon 2009). There is a crosstalk between Nrf2 and other transcription factors, amongst them the nuclear factor κ-light-chain enhancer of activated B cells NF-κB transcription factor (Wakabayashi et al. 2010). NF-κB is an important regulator of immune response, cell proliferation, and cell death. It also plays a critical role in the promotion of inflammation and is deregulated in various diseases like cancer (Dolcet et al. 2005), asthma (Janssen-Heininger et al. 2009), and atherosclerosis (Pamukcu et al. 2011). Because of its essential regulatory role not only in redox household but also in inflammation, maintenance of metabolic and protein homeostasis, the NRF2/ARE pathway is a potential drug target (Cuadrado et al. 2019). For the class A perylenequinone ATX-II, an activation of the Nrf2/ARE pathway through the oxidative properties of the compound was observed in CHO and HT29 cells by Jarolim et al. (Jarolim et al. 2017b). However, this could not be shown for ATX-I.

## Materials and methods

### Culturing and isolation of metabolites

**Fig. 2:**
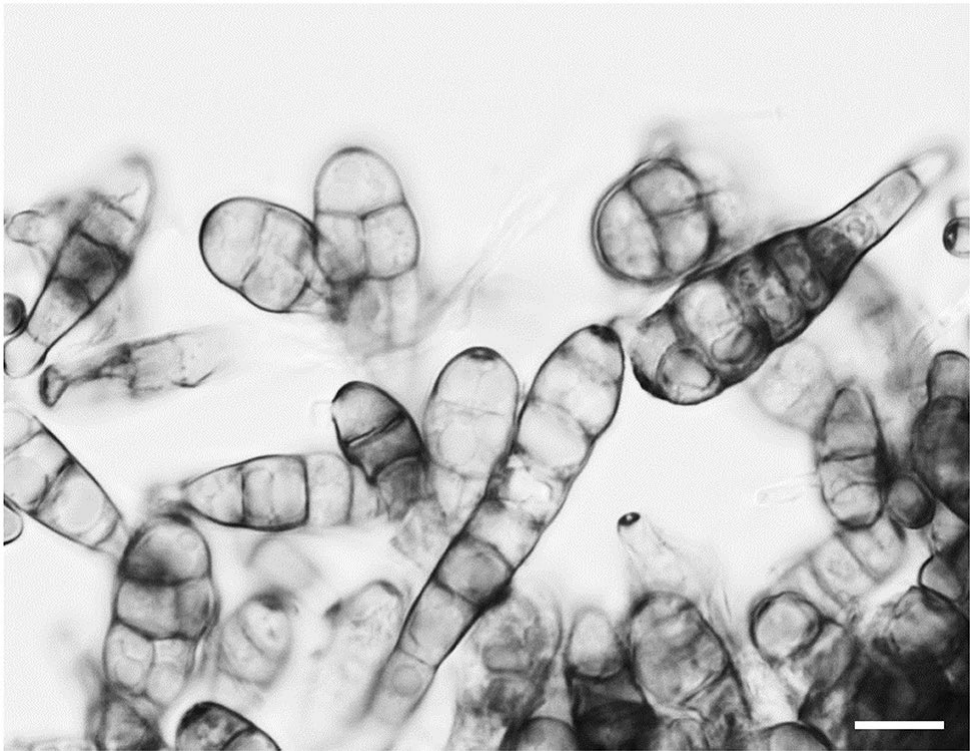
Spores of the fungus producing the compounds **1**–**3**. The cylindrical, club-shaped spores are highly segmented vertically and horizontally as it is typical for *Alternaria* sp. Sporulation was induced through exposure to UV-A radiation (Wei et al. 1985). Bar = 10 µM

*Alternaria* sp. was isolated from *Actaea spicata* (baneberry) plant material. The fungus was cultivated on standard growth medium (yeast extract 4 g/L, glucose 4 g/L, malt extract 10 g/L, 2% agar for solid media). Fungal DNA was isolated from the mycelium according to (Liu et al. 2000). Assignment to the genus *Alternaria* was made both morphologically by the typical cylindrical spore shape enlarging gradually to the end (Fig. 2) and by sequencing of the ITS1-5.8S rDNA-ITS2 region of nuclear DNA. Sequencing of the ITS region with ITS4 and ITS5 primers (White et al. 1990) revealed 99–100% similarity to different *Alternaria* species (mainly to *Alternaria rosae* and *Alternaria triticina*). Sporulation was induced by exposure to UV-A radiation (340 nm) (Wei et al. 1985).

Slices of well grown agar plates were used to inoculate 1 L liquid cultures in standard growth medium. The fungus was fermented in 2 L flasks with four baffles at room temperature and shaking (120 rpm). The fermentation was ended after 7 days of growth, and culture fluid was separated from the mycelium by filtration. Culture fluid was extracted twice with an equal volume of ethyl acetate. Phases were separated, and the organic phase was dried with Na_2_SO_4_, then concentrated in a rotary evaporator yielding 184 mg crude extract. The crude extract was purified by isocratic preparative HPLC (Agilent ZORBAX XDB-C8, 21.2 × 150 mm, 5 µm, flow 21.24 mL/min) with H_2_O + 0.1% HCO_2_H:MeCN 27:73 to yield 5.3 mg compound **1** (*t*_*R*_: 11 min), 30.7 mg ATX-1 (*t*_*R*_: 16 min), 7.3 mg compound **3** (*t*_*R*_: 25 min), and 1.2 mg compound **2** (*t*_*R*_: 28 min). The purity of the compounds was estimated to be > 95% as determined by HPLC with diode array detector and mass spectrometry (see supporting information).

### Cell culture

BEAS-2B (ATCC CRL-9609) cells were cultivated in DMEM supplemented with 10% FCS, 65 µg/mL penicillin G, and 100 µg/mL streptomycin at 37 °C and 5% CO_2_. Benzoquinone was purchased from Sigma-Aldrich (B10358).

### Cytotoxicity

Cytotoxic effects of the compounds on BEAS-2B cells were assessed after 24 h via XTT cell viability assay (Roehm et al. 1991). Briefly, 5 × 10^4^ cells/mL were seeded into 24-well plates and incubated 24 h at standard culture conditions. The medium was then removed and replaced by medium with or without the compounds. After 24 h incubation at 37 °C, 5% CO_2_, 0.5 mL of the medium was removed and replaced by 0.25 mL of prewarmed medium containing 25 µM phenazine methosulfate and 1 mg/mL XTT. Absorbance at 480 nm was measured after 3 h.

### Reporter gene assay

A triple human ARE sequence was synthesized (Sigma Aldrich) with compatible restriction enzyme sites for Sac1 and Nhe1 and cloned into a pTA-Luc backbone (Clontech) (forward 5′CATGCAGTCACAGTGACTCAGCAGAATCTGATGCAGTCACAGTGACTCAGCAGAATCTGATGCAGTCACAGTGACTCAGCAGAATCTGG 3′, reverse 5′CTAGCCAGATTCTGCTGAGTCACTGTGACTGCATCAGATTCTGCTGAGTCACTGTGACTGCATCAGATTCTGCTGAGTCACTGTGACTGCATGAGCT 3′). Plasmid DNA (50 µg) was transfected in 3.5 × 10^7^ cells/mL by electroporation (Nepagene, Nepa 21) in 400 µL of DMEM as described in the supporting information. The cells were then suspended in DMEM supplemented with 10% FCS and antibiotics at 1 × 10^6^ cells/mL and seeded into plates. After 24 h of incubation, the medium was replaced by DMEM with 10% FCS and antibiotics with and without compounds. After further incubation for 24 h, the luciferase expression was analyzed using Dual-Glo luciferase assay system (Promega) in a plate luminometer (BMG Labtech). The CXCL10 promoter (−875 to +97 relative to the transcriptional start site) reporter plasmid has been described previously (Rohr et al. 2017). BEAS-2B cells were electroporated as described above, and luciferase expression was induced with 10 ng/mL TNF-α, 10 ng/mL IFN-γ, and 5 ng/mL IL-1β.

### Dichlorodihydrofluorescein diacetate assay

A dichlorodihydrofluorescein diacetate assay was performed to detect the changes in redox status of the cells caused by the compounds. BEAS-2B cells were seeded in black 96-well plates (5000 cells/well) and incubated for 24 h under standard conditions. 2,7-Dichlorodihydrofluorescein diacetate (Cayman Chemical) was diluted in serum-free cell culture medium without phenol red (Panserin 293S, PAN-Biotech) at a final concentration of 100 µM and applied to the cells. After 30 min at 37 °C, 5% CO_2,_ the medium was replaced by Panserin 293S with or without test compounds. After 60 min incubation at 37 °C, 5% CO_2,_ the fluorescence was measured (excitation 485 nm, emission 530 nm).

### Comet assay

Comet assay was performed as previously described by Olive and Banáth with slight changes (Olive and Banáth 2006). BEAS-2B were seeded into a 6-well plate and incubated with the respective compound for 3 h. Cells were then detached mechanically with a cell scraper. After centrifugation (1000 × g, 10 min, 4 °C), cells were resuspended in cold PBS to a density of 2 × 10^4^ cells/mL. Microscope slides were prepared by pre-coating them with 1% agarose in ddH_2_O (Agarose LE, Genaxxon bioscience). The cell suspension (400 µL) was mixed with 1.4 mL of 1% agarose (40 °C) and pipetted on the microscopic slide. Cell lysis was performed under alkaline conditions in a buffer containing 1.2 M NaCl, 100 mM EDTA, 1% Triton-X 100, and 300 mM NaOH for 18–20 h at 4 °C. Before electrophoresis, slides were washed with electrophoresis buffer (30 mM NaOH, 2 mM Na_2_EDTA) three times for 20 min to remove residual salts. Electrophoresis was performed at 0.6 V/cm for 30 min. Slides were rinsed with ddH_2_O before staining with 2.5 µg/mL propidium iodide. Imaging was done with an Olympus BX53 system with MC50 Microscope Camera (Zeiss). Analysis was performed with CometScore (TriTek).

### Quantitative real-time PCR

To test for alterations in expression of selected antioxidative genes, BEAS-2B cells were seeded in plates and grown to 70% confluence. The medium was then replaced by fresh DMEM containing 10% FCS and antibiotics with and without test compounds. After incubation for 8 or 16 h, the medium was removed. Cells were washed with PBS and scraped off the plate. RNA extraction was performed using the GenUP^™^ Total RNA Kit (Biotechrabbit) according to manufacturer’s instructions. First-strand cDNA was generated using M-MLV Reverse Transcriptase (Promega) according to the manual. Relative mRNA levels were detected using 5 × HOT FIREPol^®^ EvaGreen^®^ qPCR Supermix (Solis Biodyne) and specific primers (see supporting information) using StepOnePlus real-time PCR System (Thermo Fisher Scientific). The following protocol was used for quantitative amplification: initial inactivation for 12 min at 95 °C; 15 s at 95 °C, 30 s at 56 °C, 30 s at 72 °C for 40 cycles. Relative mRNA levels were calculated using ΔΔCt (Livak and Schmittgen 2001).

To test for antioxidative properties, BEAS-2B cells were seeded in plates and grown to 70% confluence. The medium was then replaced with medium containing 30 µM benzoquinone to induce oxidative stress, and compounds were added additionally, if applicable. Cells without benzoquinone and test compounds were used as control. After 16 h, RNA isolation and quantitative real-time PCR was performed as described before.

### Western blot analysis

BEAS-2B cells were seeded into 100 mm dishes and incubated until they reached 70% confluence. The medium was then removed and replaced by medium with or without test substances. After 16 h, the medium was removed. The cells were then washed with PBS and lysed in ice cold RIPA buffer (150 mM NaCl, 50 mM Tris pH 7.4, 1% Nonidet P-40, 0.1% SDS, 0.5% sodium deoxycholate, 5 mM EDTA) supplemented with 1 × protease inhibitor cocktail (completeTM EDTA free Protease Inhibitor Cocktail, Roche). Cell debris was removed by centrifugation (8000 × g, 10 min, 4 °C) and protein content determined using the PierceTM BCA Protein Assay kit (Thermo Fisher Scientific) according to the manual. Lysates were then mixed with equal amounts of 50 mM DTT, 50 mM Na_2_CO_3_, 2.5% (w/v) SDS, and 15% (w/v) sucrose and boiled for 5 min at 95 °C. Equal amounts of protein were then separated on a 10% SDS–polyacrylamide gel and subsequently transferred to a nitrocellulose membrane. Primary antibodies used to detect protein of interest were anti-Nrf2 (MABC1556, Sigma-Aldrich, 1:2,000), anti-HMOX1 (ZRB1609, Sigma-Aldrich, 1:2,000), anti-TrxR1 (sc-28321, Santa Cruz Biotechnology, 1:500), anti-NQO1 (sc-32793, Santa Cruz Biotechnology, 1:500), anti-GAPDH (sc-47724, Santa Cruz Biotechnology, 1:1,000). Secondary antibodies used were anti-mouse (sc-516102, Santa Cruz Biotechnology, 1:10,000) and anti-rabbit HRP (A9169, Sigma-Aldrich, 1:60,000).

### Statistical analysis

Significance of quantitative real-time PCR, dichlorodihydrofluorescein diacetate (DCF-DA), and reporter gene assay results was evaluated via one sample two-sided *t*-test. Multiple testing correction was performed according to Benjamini and Hochberg (1995). Significance of comet assay was evaluated using Welch’s *t*-test after transforming the data using the logit-function. Analyses were performed using OriginPro 2021 (OriginLab Corporation) and Microsoft Excel (Microsoft Corporation).

### NMR spectroscopy

NMR spectra were recorded on a Bruker Avance-III (^1^H NMR: 600 MHz, ^13^C NMR: 151 MHz) spectrometer. All chemical shifts are referenced to the signal of the residual solvent (CD_3_OD: 3.35 ppm and 49.3 ppm for ^1^H NMR and ^13^C NMR, respectively) and reported in parts per million (ppm) relative to tetramethylsilane (TMS). For multiplicities of NMR signals, the following abbreviations were used: s = singlet, d = doublet, t = triplet, q = quartet, m = multiplet, and combinations thereof. Spectra were processed with the software MestReNova from MestrelabResearch.

### Mass spectrometry

Electrospray ionization (ESI) mass spectra were measured on an Agilent Infinity II 1200. Mixtures of water (containing 0.1% formic acid) and acetonitrile at a flow rate of 1.0 mL/min were used as eluent. An Ascentis Express C18 column from Supelco (2.7 μm particle size, 3 cm column length, 2.1 mm diameter) was used at a temperature of 40 °C. High-resolution electrospray ionization (HR-ESI) spectra were recorded on an Agilent 6545 QTOF-LC/MS with a suitable external calibrant.

### Infrared spectra

Infrared (IR) spectra were recorded on a FT-IR spectrometer (Bruker Tensor 27) with a diamond ATR unit and are reported in terms of frequency of absorption *ṽ* (cm^−1^).

### Optical activity

The optical activity of chiral compounds was determined using a Perkin-Elmer 241 MS polarimeter.

The electronic circular dichroism (ECD) spectra of compounds (+)-**1** and (+)-**3** were recorded on a JASCO J-815 spectrometer equipped with a JASCO PTC-423S/15 temperature controller at 20 °C. Each compound was measured using a scanning speed of 50 nm/min. Each measurement was repeated five times, and the five replicates were averaged. Applying the same parameters, the solvent background was recorded and subtracted from the sample measurement.

For the simulation of compound **1** and **3**, a conformational analysis was first performed (Spartan’10; Wavefunction, Inc., Irvine, CA, USA, 2009), followed by geometry refinement at DFT level. Even though both compounds are fairly rigid, several conformers in the range of 4.5 kcal/mol above the energetically lowest conformer were identified from the respective set of the conformer distribution. After calculation of the electronic excitations using time-dependent DFT and Boltzmann weighting, the ECD spectra were simulated and compared to the experimental spectra (see supporting information for computational details). All quantum mechanical calculations were performed with Gaussian 16 (Frisch et al. 2019), while the spectra comparison was conducted using SpecDis (Bruhn et al 2017).

## Results and discussion

### Structure elucidation

**Fig. 3:**
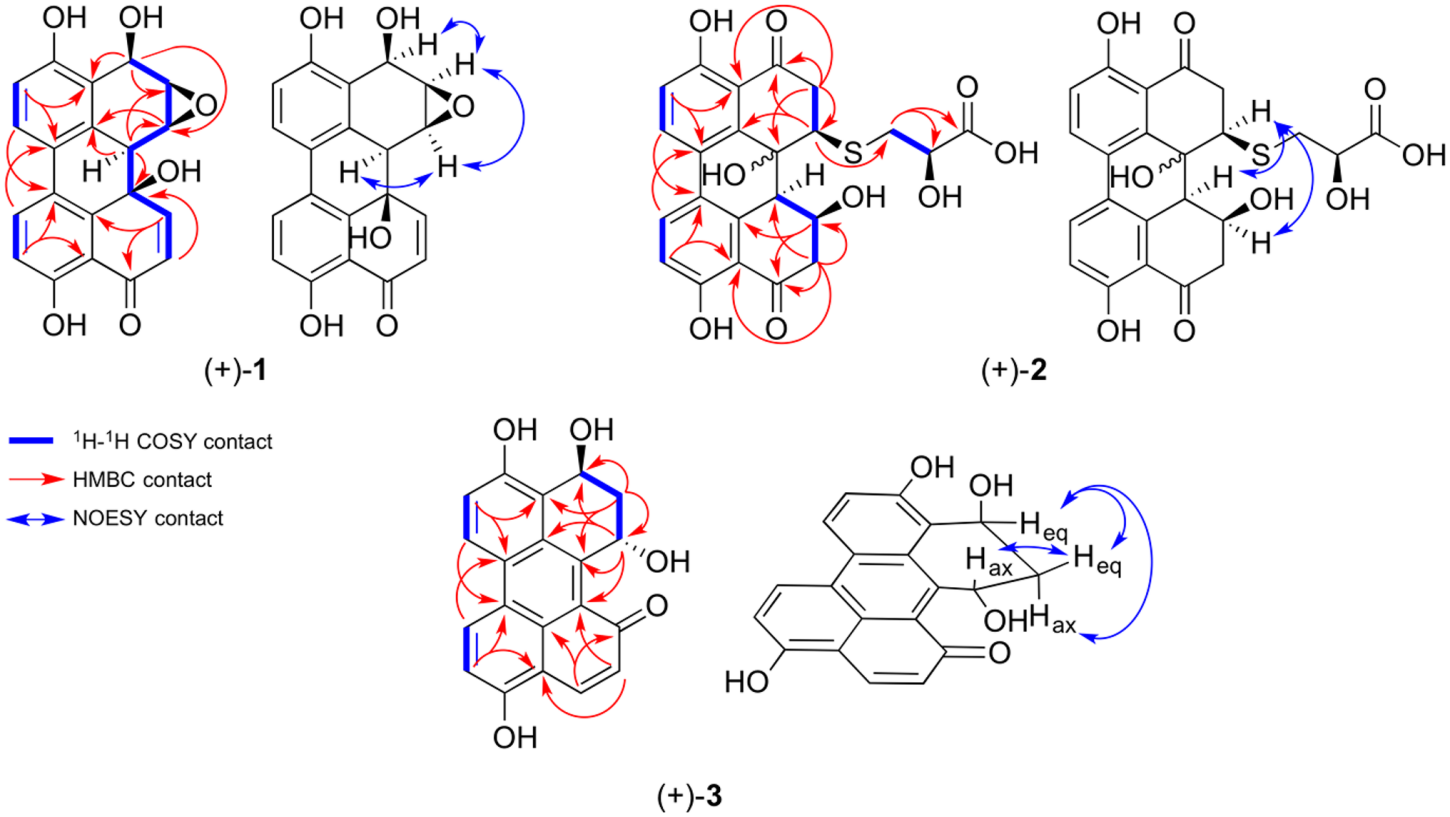
Graphic display of key ^1^H–^1^H COSY, HMBC, and NOESY contacts of compounds (+)-**1**–(+)-**3**. COSY contacts: bold blue, HMBC contacts: single headed red arrows, NOESY-contacts: double headed blue arrows

Four major compounds were isolated from the crude extract by preparative HPLC. The largest fraction proved to mainly consist of well-known altertoxin I (Stinson et al. 1982) with all analytical data matching the literature. Additionally, three further compounds (+)-**1**–(+)-**3** were isolated (Table 1; Fig. 3). The structure of (+)-**1** appears to be a reduced form of the previously described stemphyltoxin III (Arnone et al. 1986). Compounds (+)-**2** and (+)-**3** have also not been described before in literature to the best of our knowledge. Unfortunately, all attempts to crystallize compounds **1**–**3** from various solvents were unsuccessful.

**Table 1:**
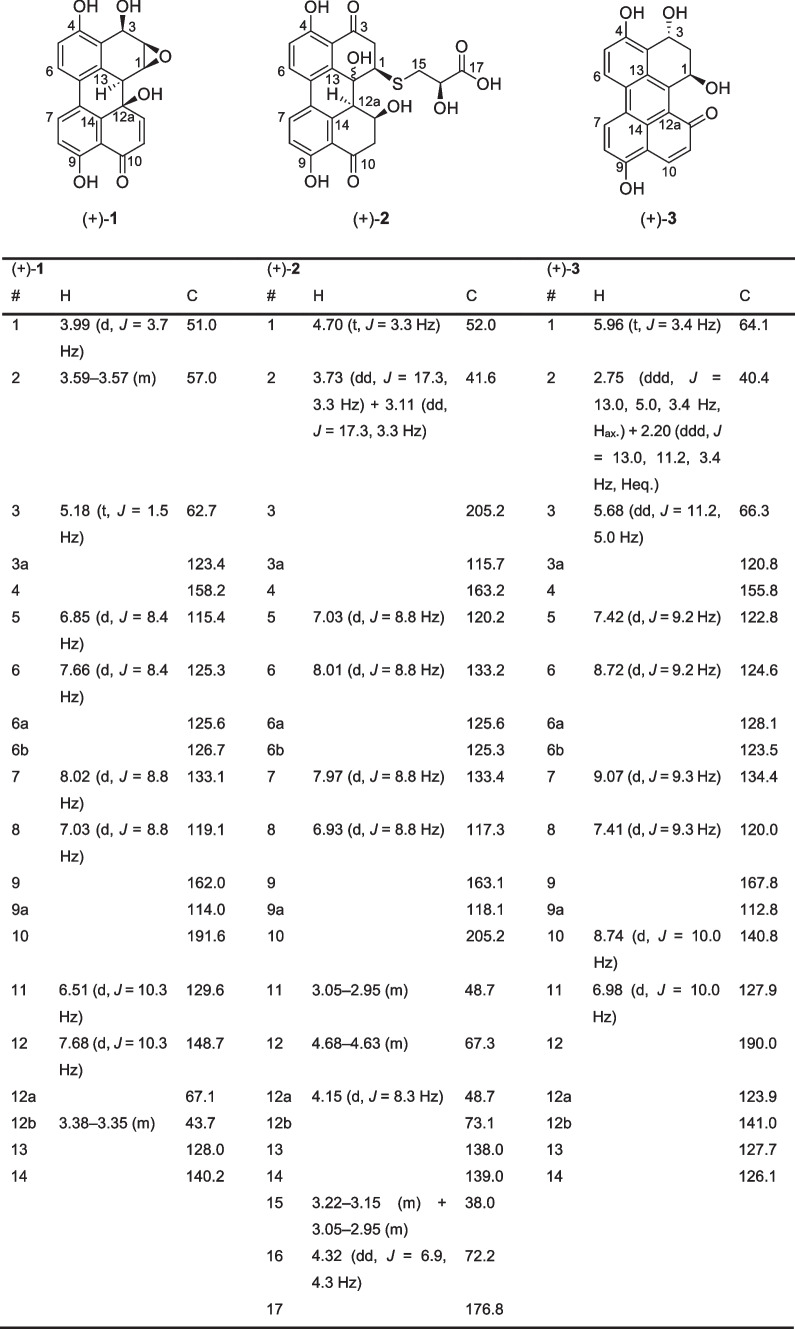
Structural formulars of compounds (+)-**1**–(+)-**3** and assignment of ^1^H (600 MHz) and ^13^C 150 MHz) signals measured in methanol-*d4* at 294 K

Compound (+)-**1** was isolated as an amorphous yellow solid. Its molecular formula was determined to be C_20_H_14_O_6_ by HR-ESI–MS (calc. for [C_20_H_14_O6–H]^−^: *m*/*z* = 349.0717, found: 349.0715), and ^1^H-,^13^C-, and 2D-NMR spectroscopy enabled the complete assignment of all carbon and proton signals. The ^13^C-, HSQC-, and HMBC-NMR spectra showed 20 carbon signals assigned to 12 aromatic carbons of which four are protonated, and one conjugated ketone carbonyl.

The COSY correlations of H-5/H-6 (6.85 ppm and 7.66 ppm) and H-7/H-8 (8.02 ppm and 7.02 ppm) along with their appearance as four doublets possessing coupling constants of 8.4 Hz and 8.8 Hz, respectively, revealed the presence of two 1,2,3,4-tetrasubstituted benzene moieties. The HMBC correlations from H-6 (7.66 ppm) to C-6b (126.7 ppm) and from H-7 (8.02 ppm) to C-6a (125.6 ppm) suggested the presence of a C-6a/C-6b linkage. Moreover, the observed HMBC correlations from H-6 (7.66 ppm) to C-4 (158.2 ppm) and from H-7 (8.02 ppm) to C-9 (162.0 ppm) revealed the presence of a 4,4′-dihydroxybiphenylic system. The COSY correlations of H-11/H-12 (6.51 ppm and 7.68 ppm) with the downfield-shifted carbon C-12 (148.7 ppm) suggests another double bond being attached to a carbonyl C-10 (191.6 ppm). The HMBC contacts from H-11 (6.51 ppm) and H-8 (7.02 ppm) to carbon C-9a (114.0 ppm) show that this conjugated ketone is attached to carbon C-9a (114.0 ppm) via the carbonyl group. Further HMBC contacts from H-11 (6.51 ppm) to the oxygenated quaternary carbon C-12a (67.1 ppm) and from H-12 (7.68 ppm) to the quaternary aromatic carbon C-14 (140.2 ppm) lead to the conclusion that one aromatic core is annealed with a cyclohexenone moiety, suggesting this compound to be also structurally related to the altertoxin family members.

The remaining signals belong to a tertiary hydrogen H-12b (3.38–3.35 ppm) and to three secondary hydrogens H-1–H-3 (3.99 ppm, 3.59–3.57 ppm, and 5.18 ppm) attached to the oxygenated carbons C-1–C-3 (51.0 ppm, 57.0 ppm, and 62.7 ppm). However, the molecular formula has only two more oxygen atoms, thereby indicating the presence of an epoxide functionality. The HMBC contacts from H-12b (3.38–3.35 ppm) and H-1 (3.99 ppm) to quaternary aromatic carbon C-13 (128.0 ppm) together with the HMBC contacts from H-3 (5.18 ppm) to quaternary aromatic carbon C-3a (123.4) and oxygenated carbons C-2 and C-3 (57.0 ppm and 62.7 ppm) led to the complete assignment of structure (+)-**1**.

The relative stereochemistry was deduced by NOESY-NMR spectroscopy. NOESY contacts from H-12b (3.38–3.35 ppm) to H-1 (3.99 ppm), from H-1 (3.99 ppm) to H-2 (3.59–3.57 ppm), and from H-2 (3.59–3.57 ppm) to H-3 (5.18 ppm) indicate that protons H-1–H-3 (3.99 ppm, 3.59–3.57 ppm and 5.18 ppm) and H-12b (3.38–3.35 ppm) share the same side of the molecule. The absolute configuration of compound (+)-**1** was tentatively determined to (1*S*,2*R*,3*R*,12a*R*,12b*S*) by comparing measured and calculated electronic circular dichroism (ECD) spectra (see supporting information for detailed results).

Compound (+)-**2** was isolated as an amorphous yellow solid. Its molecular formula was determined to be C_23_H_20_O_**9**_S by HR-ESI–MS (calc. for [C_23_H_20_O_9_S–H]^−^: *m*/*z* = 471.0755, found: 471.0739), and ^1^H-, ^13^C-, and 2D-NMR spectroscopy enabled the complete assignment of all carbon and proton signals. The ^13^C-, HSQC-, and HMBC-NMR spectra showed 23 carbon signals assigned to three methylenes, 12 aromatic carbons of which four are protonated, two conjugated ketone carbonyls, and one carboxyl carbon.

The COSY correlations of H-5/H-6 (7.03 ppm and 8.01 ppm) and H-7/H-8 (7.97 ppm and 6.93 ppm) along with their appearance as four doublets possessing a coupling constant of 8.8 Hz revealed the presence of two 1,2,3,4-tetrasubstituted benzene moieties. The HMBC correlations from H-6 (8.01 ppm) to C-6b (125.3 ppm) and from H-7 (7.97 ppm) to C-6a (125.6 ppm) suggested that there is a C-6a/C-6b linkage. Moreover, the observed HMBC correlations from H-6 (8.01 ppm) to C-4 (163.2 ppm) and from H-7 (7.97 ppm) to C-9 (163.1 ppm) revealed the presence of a 4,4′-dihydroxybiphenylic system. The observed HMBC correlations from H-2 (3.73 ppm and 3.09 ppm) to C-1 (52.0 ppm), C-12b (73.1 ppm), C-3a (115.7 ppm) and carbonyl C-3 (205.2 ppm) alongside the HMBC correlations from H-11 (3.05–3.01 ppm) to C-12a (48.6 ppm), C-12 (67.3 ppm), C-9a (118.1 ppm), and carbonyl C-10 (205.1 ppm) lead to the conclusion that the core structure of compound **2** is also similar to that of the altertoxin natural products.

The chemical shifts of C-12 (67.3 ppm) and C-12b (73.1) furthermore indicate hydroxyl groups at this position. The observed chemical shift of C-1 (52.0 ppm) together with the HRMS experiment and the HMBC correlation of H15 (3.18 ppm and 3.02–2.98 ppm) to C-1 (52.0) suggests that the remaining part of the molecule is linked via a thioether bridge at C-1 (52.0 ppm). The COSY correlation between H-15 (3.18 ppm and 3.02–2.98 ppm) and H-16 (4.32 ppm) alongside their HMBC contacts to the carboxyl C-17 (176.8 ppm) unambiguously shows the presence of a 3-mercaptolactate moiety.

The relative stereochemistry was partly deduced by NOESY-NMR spectroscopy. Strong NOESY contacts from H-1 (4.70 ppm) to H-12 (4.69–4.62 ppm) and H-12a (4.15 ppm) indicate that these protons show to the same side of the molecule (see supporting information for detailed results). As compound (+)-**2** was isolated from a fungus, we assumed the stereogenic center at C-16 (72.2 ppm) to possess a *R*-configuration, as lower fungi predominantly produce d-3-mercaptolactate (Meng et al. 2013). A related compound was isolated in 2020 in the Rychlik group from *Alternaria alternata*; however, the orientation of the hydroxy group was not determined here either (Gotthardt 2020). With the protons of the 1-, 12-, and 12a-position showing to the same side of the molecule but the orientation of the hydroxyl group at the 12b-position remaining unclear, the absolute configuration of (+)-**2** could not unequivocally be determined.

Compound (+)-**3** was isolated as an amorphous orange-red solid. Its molecular formula was determined to be C_20_H_14_O_5_ by HR-ESI–MS (calc. for [C_20_H_14_O_5_–H]^−^: *m*/*z* = 333.0768, found: 333.0763) and intensive ^1^H-, ^13^C-, and 2D-NMR spectroscopy enabled the complete assignment of all carbon and proton signals. The ^13^C-, HSQC-, and HMBC-NMR spectra showed 20 carbon signals assigned to one methylene, 14 aromatic carbons of which four are protonated, and one conjugated ketone carbonyl.

The COSY correlations of H-5/H-6 (7.42 ppm and 8.72 ppm) and H-7/H-8 (9.07 ppm and 7.41 ppm) along with their appearance as four doublets possessing coupling constant of 9.2 Hz and 9.3 Hz, respectively, revealed the presence of two 1,2,3,4-tetrasubstituted benzene moieties. The HMBC correlations from H-6 (8.72 ppm) to C-6b (123.5 ppm) and from H-7 (9.07 ppm) to C-6a (128.1 ppm) suggested that there is a C-6a/C-6b linkage. Moreover, the observed HMBC correlations from H-6 (8.72 ppm) to C-4 (155.8 ppm) and from H-7 (9.07 ppm) to C-9 (167.8 ppm) revealed the presence of a 4,4′-dihydroxybiphenylic system. The observed HMBC correlations from H-2 (2.75 ppm and 2.20 ppm) and H-3 (5.68 ppm) to C-3a (115.7 ppm) and the downfield shifted carbons C-1 (64.1 ppm) and C-3 (66.3 ppm), alongside the COSY correlations from H-2 (2.75 ppm and 2.20 ppm) to both, H-1 (5.96 ppm) and H-3 (5.68 ppm), lead to the conclusion that a 1,3-propanediol chain can only be attached to carbon C-3a (115.7 ppm) via carbon C-3 (66.3 ppm).

The COSY correlation of H-10/H-11 (8.74 ppm and 6.98 ppm) and the HMBC contacts from H-10 (8.74 ppm) to C-12 (190.0 ppm) and C-14 (126.1 ppm), alongside the downfield shift of C-10 (140.8), unambiguously show the presence of an α,β-unsaturated ketone attached to carbon C-9a (112.8 ppm) via carbon C-10 (140.8 ppm). Four further HMBC contacts from H-11 (6.98 ppm) to aromatic carbon C-12a (123.9) and from H-1 (5.96 ppm) to aromatic carbons C-12a (123.9 ppm), C-12b (141.0 ppm), and C-13 (127.6 ppm) indicated that compound (+)-**3** share a partially saturated perylenone core structure.

The relative stereochemistry was deduced by the coupling constants of H-1 (5.96 ppm) and H-3 (5.68 ppm). While H-1 (5.96 ppm) appears as a pseudo-triplet with a coupling constant of 3.4 Hz and therefore likely occupies an equatorial position, H-3 (5.68 ppm) forms a clean doublet of doublets with coupling constants of 11.2 Hz and 5.0 Hz, thereby indicating an axial position of H-3. Consequently, the hydroxyl groups at C-1 (64.1 ppm) and C-3 (66.3 ppm) take up a *trans* relationship. The absolute configuration of (+)-**3** was tentatively determined as (1*R*,3*R*) by means of ECD and quantum mechanical calculations as previously described (see the supporting information for detailed results).

### Biological activities

During a screening for biologically active compounds, we found that the crude extracts and extract fractions of *Alternaria* spec. showed effects on an ARE-transcriptional reporter carrying a triple ARE. The reporter plasmid was transiently transfected into BEAS-2B cells. As positive control, *p*-benzoquinone was applied to the cells which induces reactive oxygen species formation. Subjecting BEAS-2B to benzoquinone has been previously shown to induce Nrf2 expression and ARE reporter activity leading to elevated levels of Nrf2-dependent proteins (Rubio et al. 2011). After activity guided purification of three compounds, the same reporter was used to confirm the activity observed in the crude extract and its fractions. The fungus produced several perylene quinones; however, compound (+)-**2** was only obtained in minute amounts and could therefore not be subjected to further biological testing.

**Fig. 4:**
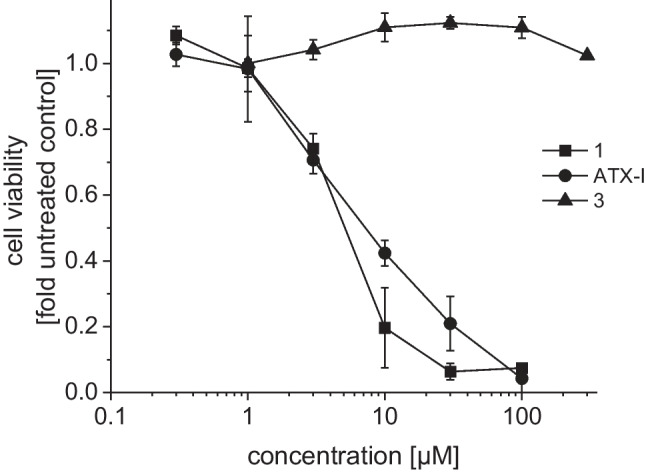
Cytotoxic effects of compounds **1**, ATX-I, and **3** on BEAS-2B cells. Cytotoxicity was analyzed by XTT cell viability assay. Cells were incubated with compounds for 24 h. Untreated BEAS-2B cells were used as control. Data are shown as mean ± SEM from three independent replicates

The cytotoxicity of the isolated compounds was assessed through an XTT cell viability assay for BEAS-2B cells. The strongest effect was observed after application of compound (+)-**1** (LC_50_ = 3.8 ± 0.13 µM), ATX-I also showed strong cytotoxic effects (LC_50_ = 6.43 ± 0.86 µM), while compound (+)-**3** had no effects on the viability of the cells (Fig. 4). The effect of higher concentrations could not be analyzed because of the limited solubility of the compounds in the cell culture medium.

**Fig. 5:**
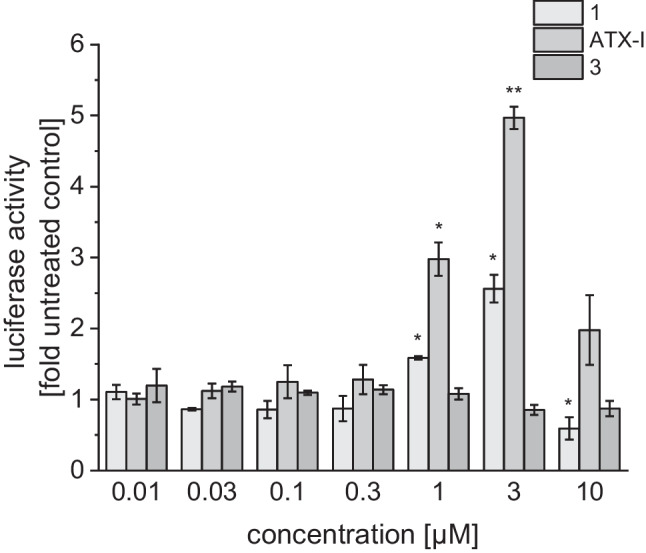
Effect of compounds **1**, ATX-I, and **3** on ARE reporter gene activity in BEAS-2B cells. Cells were transiently transfected with an ARE dependent luciferase reporter construct by electroporation. BEAS-2B treated with 30 µM benzoquinone and untreated cells were used as control. Data are shown as mean ± SEM from three independent replicates

Compound (+)-**1**, ATX-I, and compound (+)-**3** showed modulation of the ARE reporter activity albeit with different specificity. As a control 30 µM benzoquinone was applied to induce the ARE reporter. Benzoquinone mediates the release of Nrf2 from KEAP and subsequently the induction of ARE-dependent gene expression in BEAS-2B cells (Rubio et al. 2011). Upon treatment with benzoquinone, the luciferase levels increased to 2.8 ± 0.4-fold compared to the untreated control (Fig. 5). ATX-I and (+)-**1** displayed concentration-dependent activation of the triple ARE transcriptional reporter. ATX-I exhibited the strongest effect to the ARE reporter with an induction of 5.0 ± 0.15-fold at 3 µM, while (+)-**1** induced to levels of 2.6 ± 0.19-fold at the same concentration. Application of higher concentrations of ATX-I and (+)-**1** led to a massive drop of luciferase activity, presumably due to cytotoxic effects. Induction of ARE by ATX-I was previously tested in reporter gene assays by Jarolim et al. (2017a) in CHO cells, where application of up to 5 µM did not lead to significant induction of luciferase levels (Jarolim et al. 2017b). In contrast, we observed a significant induction of Nrf2/ARE-dependent luciferase expression after application of 3 µM ATX-I in the bronchial epithelial cell line BEAS-2B which may be due to a higher sensitivity to the compound and/or differences in the expression and activation of NRF2 in various normal and transformed cell lines (Kitamura and Motohashi 2018).

**Fig. 6:**
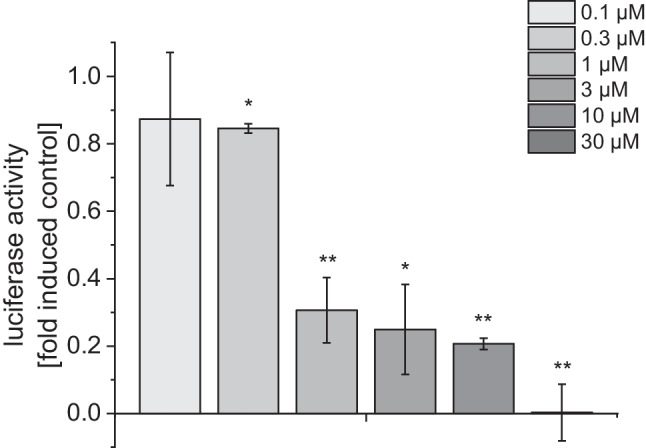
Effect of compound **3** on ARE reporter activity after induction of oxidative stress with 30 µM benzoquinone. BEAS-2B cells treated with 30 µM benzoquinone were used as control. Untreated cells were used to determine basal activity of ARE construct. Data are shown as mean ± SEM from three independent replicates

**Fig. 7:**
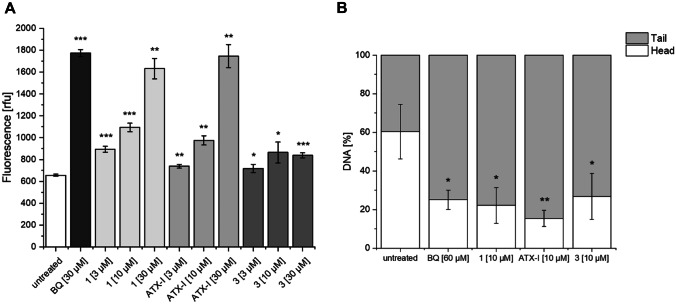
Influence of **1**, ATX-I, and **3** on redox state of BEAS-2B cells. **A** 2,7-Dichlorodihydrofluorescein diacetate was applied to the cells; after 30 min, it was replaced by serum-free medium with and without test compounds. After 60 min, the fluorescence was measured. Data are shown as mean ± SEM from three independent replicates. *p < 0.05, **p < 0.01, ***p < 0.001. **B** BEAS-2B cells were treated with the indicated compounds for 3 h. After cell lysis, alkaline comet assay was performed. Percentage of DNA in head and tail was analyzed using CometScore. Data are shown as mean ± SEM from three independent replicates. * < 0.05, ** < 0.01

Interestingly compound (+)-**3** showed no significant induction of NRF2/ARE mediated luciferase expression in the reporter gene assay but strongly inhibited ARE-dependent reporter activity in BEAS-2B cells after induction of the antioxidative response with 30 µM benzoquinone (Fig. 6). The benzoquinone-induced luciferase expression was reduced by 70% compared to the untreated control at a concentration of 1 µM of compound (+)-**3**. Various synthetic and natural activators of the NRF2/ARE pathway including altertoxin II have been found to suppress production of pro-inflammatory cytokines by interfering with NF-κB and interferon signaling (Cuadrado et al. 2019; Del Favero et al. 2020; Ryan et al. 2022). We therefore investigated the effect of the isolated compounds on the TNF-α/IL-1β/IFN-γ inducible CXCL10 transcriptional reporter as a pro-inflammatory marker gene in transiently transfected BEAS-2B cells. The inducible expression of luciferase therefore reflects the cooperative induction of *cxcl10* mRNA expression by Stat1, NF-κB, and IRF3 transcription factors (Tamassia et al. 2007). Stimulation of transfected cells with 10 ng/mL TNF-α, 5 ng/mL IL-1β, and 10 ng/mL IFN-γ increased the luciferase activity sixfold compared to the uninduced control. None of the isolated compounds significantly affected the inducible expression of the CXCL10 promoter activity up to the highest concentrations tested (0.3 µM for ATX-I and (+)-**1**, 3 µM for (+)-**3**), indicating that the compounds do not display anti-inflammatory properties (see supplementary information). To investigate the oxidative properties of the compounds, we used a dichlorodihydrofluorescein diacetate assay as described in the "Materials and methods" section. The fluorescence measurement was performed after 60 min. As positive control 30 µM *p*-benzoquinone was applied to the BEAS-2B cells which led to significantly elevated fluorescence levels compared to the untreated control. Compound (+)-**1**, ATX-I, and (+)-**3** were applied in concentrations of 3 µM, 10 µM, and 30 µM (Fig. 7A). Application of compound (+)-**1** and ATX-I led to a strong increase of fluorescence in a concentration dependent manner, whereas application of (+)-**3** only slightly affected fluorescence levels in comparison to the untreated control. We assume that the rather small increase in fluorescence after application of up to 30 µM of (+)-**3** may be caused by the reaction of the compound with the DCF radical which is generated during the reaction of DCFH-DA to the fluorescent DCF and can lead to self-propagating redox cycling (Kalyanaraman et al. 2012). Also, with this assay, only the total redox state of the cells can be detected. DCFH-DA reacts with a variety of reactive species like hydroxyl radicals, hypochlorous acid, and nitrogen dioxide leading to a rather low specificity (Kalyanaraman et al. 2012). However, regardless of these limitations, it can be assumed that compound (+)-**1** and ATX-I cause a significant shift in the redox state in BEAS-2B cells.

The induction of oxidative stress by various *Alternaria* perylenquinones such altertoxin II and stemphyltoxin III has been implicated in the DNA-damaging properties of these compounds (Aichinger et al. 2021). To analyze possible DNA damage caused by application of the isolated compounds, a comet assay was conducted. The percentage of DNA in head and in tail is visualized in Fig. 7B. Application of 60 µM benzoquinone and either 10 µM compound (+)-**1**, ATX-I, and compound (+)-**3** resulted in a significant increase in tail DNA (BQ: 75 ± 5%, (+)-**1**: 77.8 ± 9.2%, ATX-I: 84.6 ± 4.2%, (+)-**3**: 73.2 ± 11.9%), indicating increased rates of double and single strand breaks.

**Fig. 8:**
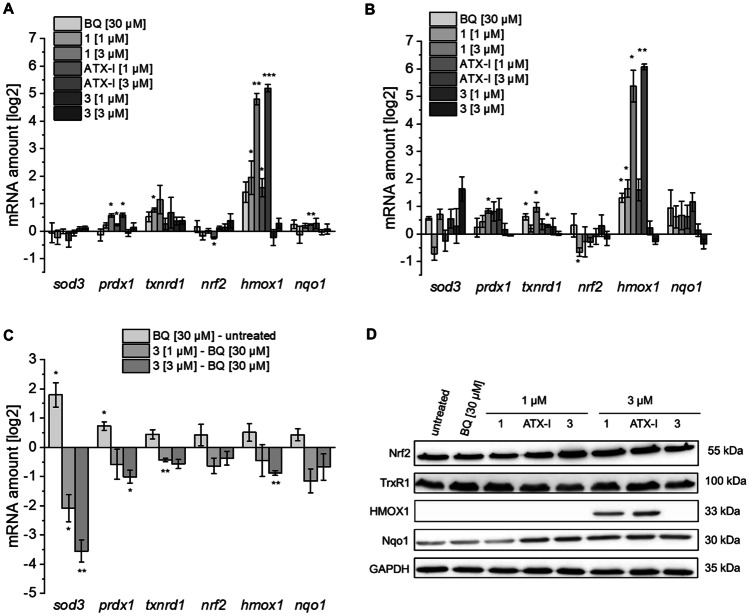
Effects of compounds **1**, ATX-I, and **3** on mRNA expression and protein levels of selected ARE dependent genes in BEAS-2B cells. The cells were incubated with the compounds for 8 h (**A**) and 16 h (**B**), respectively. **C** Effect of compound **3** on mRNA expression levels of selected ARE dependent genes in BEAS-2B cells after induction of oxidative stress with benzoquinone. The cells were incubated with the compound for 16 h. All mRNA levels refer to *gapdh* as housekeeping control. Data are shown as mean ± standard deviation from three independent replicates. Asterisks indicate *q*-values of * < 0.05, and ** < 0.01. **D** Western blot analyses of selected anti-oxidative proteins. BEAS-2B cells were treated with indicated compounds for 16 h. GAPDH was detected as a housekeeping control

Since all three tested compounds affected the triple ARE transcriptional reporter, we investigated their effect on the expression levels of selected NRF2/ARE-induced genes via quantitative real-time PCR (Ma 2013). Relative mRNA amounts were calculated in relation to expression of a housekeeping gene (*gapdh*) and compared to the untreated control. As shown in Fig. 8A–B, benzoquinone, compound (+)-**1**, and ATX-I strongly upregulated the mRNA levels of heme oxygenase-1 (*hmox1*), regardless of the incubation time. After 16 h, a slight upregulation of mRNA levels for NAD(P)H dehydrogenase (quinone) 1 (*nqo1*) could be observed. Application of compound (+)-**3** had almost no effect on the selected mRNA levels. After 16 h incubation with 3 µM of (+)-**3**, *sod3* mRNA levels were slightly elevated: To assess whether compound (+)-**3** inhibits NRF2/ARE mediated mRNA transcription, real-time PCR analyses were performed with cells co-treated for 16 h with compound (+)-**3** and 30 µM benzoquinone to induce oxidative stress. Compared to the untreated control, benzoquinone slightly induced the levels of all tested mRNAs. Application of compound (+)-**3** decreased the expression of all mRNAs analyzed starting at 1 µM with the strongest suppression of superoxide dismutase 3 (*sod3*) gene expression (Fig. 8C), an extracellular antioxidant enzyme which plays an essential role in the pathogenesis of inflammatory diseases and cancer (Nguyen et al. 2020; O´Leary et al. 2021).

To analyze the effect of the isolated compounds on levels of selected proteins involved in antioxidative response, cells were treated with compound (+)-**1**, ATX-I, and compound (+)-**3** for 16 h in comparison with benzoquinone. Cells were lysed, and protein extracts were then subjected to SDS-PAGE and western blotting. While there was no significant change in Nrf2 and Nqo1 levels, a strong upregulation of HMOX1 protein levels after treatment with 3 µM of compound (+)-**1** and ATX-I (Fig. 8D) could be detected. Accordingly, the upregulation of the *hmox1* mRNA levels observed during quantitative real-time PCR was also confirmed at the protein level. HMOX1 is a highly inducible enzyme which primarily functions in in heme catabolism, where it catalyzes the breakdown of heme into iron, biliverdin, and CO. Besides, it is involved in anti-oxidative and anti-inflammatory responses and is known to be upregulated by most cells in reaction to various stress conditions (Campbell et al. 2021). Strong upregulation of HMOX1 was also observed in many human cancers such as lung and gastric cancer where it plays an important role in cancer progression and resistance to anti-tumor therapy (Degese et al. 2012; Yin et al. 2012). In case of the compounds (+)-**1** and ATX-I, the induction of HMOX1 expression presumably is a coping mechanism due to the generation of oxidative stress.

In summary, we identified three new perylenequinones compounds (+)-**1**–**3** along with the previously isolated altertoxin I (ATX-1) from fermentations of an *Alternaria* species. The minor metabolite compound (+)-**2** contains an esterified 3-mercaptolactate group. Several sulfur-containing polyketides from fungi have been described so far which include pandangolide 3 (also bearing a mercaptolactate moiety) and pandangolide 4, where a sulfide bridge connects two macrocyclic polyketides, isolated from *Cladosporium herbarum* and the related thiocladospolides A-D from *Cladosporium cladosporoides* (Jadulco et al. 2001; Zhang et al. 2019). Also, the sulfur-containing curvularin derivatives sumalarins A-C, previously isolated from fermentations of *Penicillium sumatrense*, contain the same mercaptolactone moity (Meng et al. 2013). Recently, it has been shown that the spontaneous condensation of 10,11-dehydrocurvularin with 3-mercaptopyruvate originating from L-cysteine to cyclothiocurvularin followed by further oxidation to cyclosulfoxicurvularin may be a detoxification reaction under stress conditions (Castro et al. 2016). Therefore, it seems conceivable that the production of the mercaptolactate-containing compound (+)-**2** also represents a detoxification process by the fungus to reduce toxicity of the altertoxins. Compound (+)-**1** and altertoxin I display cyctotoxic effects, oxidative properties, induce DNA damage, activate the NRF2/ARE pathway, and strongly upregulate heme oxygenase 1 expression on mRNA and protein level in BEAS-2B cells. Although the DNA-damaging properties of some altertoxins such as altertoxin II or stemphyltoxin III has been attributed to the occurrence the reactive epoxy moiety forming DNA-adducts (Soukup et al. 2020), we observed similar DNA-damaging properties of compound (+)-**1**, bearing an epoxy group, and altertoxin I in BEAS-2B cells. In contrast to compound (+)-**1** and altertoxin I, compound (+)-**3** exhibited no significant cytotoxicity and antagonized benzoquinone induced NRF2/ARE-dependent luciferase expression in BEAS-2B cells. In addition, compound (+)-**3** strongly inhibited ARE/NRF2 dependent *sod3* mRNA levels in benzoquinone induced BEAS-2B cells. These results indicate that compound (+)-**3** acts as a transcriptional inhibitor of NRF2/ARE signaling but the exact mode of action still has to be determined. In contrast to earlier reports for ATX II from Del Favero et al. (2020), no anti-inflammatory activity was observed after treatment with the tested Alternaria toxins in BEAS-2B cells using a CXCL-10 promoter dependent transcriptional reporter (see supplementary information).

## Supplementary Information

Below is the link to the electronic supplementary material.

Supplementary file1 (DOCX 10653 KB)

## Abbreviations

ATX: Altertoxin
PDT: Photodynamic therapy
Nrf2: Nuclear factor erythroid-derived 2
KEAP1: Kelch-like ECH-associated protein 1
ARE: Antioxidant response element
ROS: Reactive oxygen species
NF-κB: Nuclear factor κ-light-chain enhancer of activated B cells
ITS: Internal transcribed spacer
DMEM: Dulbecco’s modified eagle medium
FCS: Fetal calf serum
DCFH-DA: Dichlorodihydrofluorescein diacetate
