# Supplementary material for: Structure elucidation and biological activities of perylenequinones from an Alternaria species
Source: Mycotoxin Res. 2023 Jun 23;39(3):303–16. doi: 10.1007/s12550-023-00495-1 (PMC10393905; doi:10.1007/s12550-023-00495-1)
Supplement: Supplementary file 1 — Supplementary file1 (DOCX 10653 KB) [file 12550_2023_495_MOESM1_ESM.docx]

SUPPLEMENTARY MATERIAL

Structure elucidation and biological activities of perylenequinones from an *Alternaria* species

Anna Kiefer^1^, Marcel Arnholdt^1^, Viktoria Grimm^1^, Leander Geske², Jonathan Groß², Nina Vierengel², Till Opatz²^,§^, Gerhard Erkel^1,§^

Author’s institution/affiliation

^1^Molecular Biotechnology & Systems Biology, RPTU, Paul-Ehrlich-Straße 23, D-67663 Kaiserslautern, Germany

^2^Department of Chemistry, Johannes Gutenberg-University, Duesbergweg 10-14, D-55128 Mainz, Germany

^§^Corresponding authors: Gerhard Erkel (erkel@bio.uni-kl.de); Till Opatz (opatz@uni-mainz.de)

Electroporation parameters

|  | Voltage | Pulse Length [msec] | Pulse Interval [msec] | Number of Pulses | Decay Rate [%] | Polarity |
| --- | --- | --- | --- | --- | --- | --- |
| Poring Pulse | 220 | 5 | 50 | 2 | 10 | + |
| Transfer Pulse | 20 | 50 | 50 | 5 | 40 | +/- |

Quantitative real time PCR Primer

| SOD3 (NM_003102.4) |  |
| --- | --- |
| Sense | ACGCTGGCGAGGACGACCTG |
| Antisense | GCTTCTTGCGCTCTGAGTGCTC |
| PRDX1 (NM_181696.3) |  |
| sense | CTGCCAAGTGATTGGTGCTTCTG |
| antisense | AATGGTGCGCTTCGGGTCTGAT |
| TXNRD1 (NM_182743.3) |  |
| Sense | GTTACTTGGGCATCCCTGGTGA |
| Antisense | CGCACTCCAAAGCGACATAGGA |
| NRF2 (NM_006164.5) |  |
| Sense | CACATCCAGTCAGAAACCAGTGG |
| Antisense | GGAATGTCTGCGCCAAAAGCTG |
| HMOX1 (NM_002133.3) |  |
| Sense | ATGACACCAAGGACCAGAGC |
| Antisense | GTGTAAGGACCCATCGGAGA |
| NQO1 (NM_000903.3) |  |
| Sense | CAGTGGTTTGGAGTCCCTGCC |
| Antisense | TCCCCGTGGATCCCTTGCAG |
| Cyp1A1 (NM_000499) |  |
| Sense | GATTGAGCACTGTCAGGAGAAGC |
| Antisense | ATGAGGCTCCAGGAGATAGCAG |
| Cyp1A2 (NM_000761) |  |
| Sense | TCATCCTGGAGACCTTCCGACA |
| Antisense | GCCACTGGTTTACGAAGACACAG |
| GAPDH (NM_002046) |  |
| Sense | CCTCCGGGAAACTGTGG |
| Antisense | AGTGGGGACACGGAAG |

**1. Experimental data:**

(1*S*,2*R*,3*R*,12a*R*,12b*S*)-Alterperylenepoxide A isomer, (+)-**1**): Yellow solid. **^1^H NMR (600 MHz, CD_3_OD):** δ [ppm] = 8.02 (d, *J* = 8.8 Hz, 1H), 7.68 (d, *J* = 10.3 Hz, 1H), 7.66 (d, *J* = 8.4 Hz, 1H), 7.03 (d, *J* = 8.8 Hz, 1H), 6.85 (d, *J* = 8.4 Hz, 1H), 6.51 (d, *J* = 10.3 Hz, 1H), 5.18 (t, *J* = 1.5 Hz, 1H), 3.99 (d, *J* = 3.7 Hz, 1H), 3.59–3.57 (m, 1H), 3.38–3.35 (m, 1H). **^13^C NMR (151 MHz, CD_3_OD):** δ [ppm] = 191.7, 161.5, 158.2, 148.7, 140.5, 133.1, 129.6, 127.7, 126.7, 125.7, 125.3, 119.1, 115.4, 114.0, 67.2, 60.5, 54.7, 43.7. **HR-ESI-MS (neg):** found m/z = 349.0715, calc. m/z = 349.0717. **LC-ESI-MS (neg):** found m/z = 349.0, calc. m/z = 349.1. **IR**: 𝜈̅ [cm^–1^] = 3404, 2922, 2852, 1625, 1460, 1395, 1232, 1078, 1039. ${\mathbf{[}\boldsymbol{\alpha}\mathbf{]}}_{D}^{21}$= +230.0 (*c* = 0.26; MeOH).

****Compound **2**: Yellow solid. **^1^H NMR (600 MHz, CD_3_OD):** δ [ppm] = 8.01 (d, *J* = 8.8 Hz, 1H), 7.97 (d, *J* = 8.8 Hz, 1H), 7.03 (d, J = 8.8 Hz, 1H), 6.93 (dd, *J* = 8.8, 1.0 Hz, 1H), 4.70 (t, *J* = 3.3 Hz, 1H), 4.66 (ddd, *J* = 9.8, 8.3, 5.2 Hz, 1H), 4.32 (dd, *J* = 6.9, 4.3 Hz, 1H), 4.15 (d, *J* = 8.3 Hz, 1H), 3.73 (dd, *J* = 17.3, 3.3 Hz, 1H), 3.22–3.15 (m, 1H), 3.09 (dd, *J* = 17.4, 3.3 Hz, 1H), 3.05–2.95 (m, 3H). **^13^C NMR (151 MHz, CD_3_OD):** δ [ppm] = 205.2, 205.2, 163.2, 163.1, 139.0, 138.0, 133.4, 133.2, 125.6, 125.3, 120.2, 118.1, 117.3, 115.7, 73.1, 72.2, 67.3, 52.0, 41.6, 38.0. **HR-ESI-MS (neg):** found m/z = 471.0739, calc. m/z = 471.0755. **LC-ESI-MS (neg):** found m/z = 471.1, calc. m/z = 471.1. **IR**: 𝜈̅ [cm^–1^] = 3407, 2925, 1641, 1461, 1357, 1242, 1199, 1082, 1040. ${\mathbf{[}\boldsymbol{\alpha}\mathbf{]}}_{D}^{21}$= +110.8 (*c* = 0.76; MeOH).

(10*R*,12*R*)-Compound (+)-**3**: Orange-red solid. **^1^H NMR (600 MHz, CD_3_OD):** δ [ppm] = 9.07 (d, *J* = 9.3 Hz, 1H), 8.74 (d, *J* = 10.1 Hz, 1H), 8.72 (d, *J* = 9.3 Hz, 2H), 7.42 (d, *J* = 9.1 Hz, 1H), 7.41 (d, *J* = 9.2 Hz, 1H), 6.98 (d, *J* = 10.0 Hz, 1H), 5.96 (t, *J* = 3.4 Hz, 1H), 5.68 (dd, *J* = 11.2, 5.0 Hz, 1H), 2.75 (dt, *J* = 13.0, 4.4 Hz, 2H), 2.20 (ddd, *J* = 12.9, 11.3, 3.1 Hz, 2H). **^13^C NMR (151 MHz, CD_3_OD):** δ [ppm] = 190.0, 170.2, 167.8, 155.8, 141.0, 140.8, 134.4, 128.1, 127.9, 127.6, 126.1, 124.6, 123.9, 123.5, 122.8, 120.8, 119.9, 112.8, 66.3, 64.1, 40.4. **HR-ESI-MS (neg):** found m/z = 333.0763, calc. m/z = 333.0768. **LC-ESI-MS (neg):** found m/z = 333.1, calc. m/z = 333.1. **IR**: 𝜈̅ [cm^–1^] = 3356, 2920, 2851, 1624, 1516, 1467, 1390, 1356, 1234, 1175, 1046. ${\mathbf{[}\boldsymbol{\alpha}\mathbf{]}}_{D}^{21}$= +149.9 (*c* = 0.41; MeOH).

1. **Spectra**

Figure 1 ^1^H NMR (600 MHz, CD_3_OD): Compound (+)-**1**.

Figure 2 ^13^C NMR (150 MHz, CD_3_OD): Compound (+)-**1**.

Figure 3 ^1^H–^1^H-COSY (CD_3_OD): Compound (+)-**1**.

Figure 4 ^1^H–^13^C HSQC (CD_3_OD): Compound (+)-**1**.

Figure 5 ^1^H–^13^C HMBC (CD_3_OD): Compound (+)-**1**.

Figure 6 ^1^H–^1^H-NOESY (CD_3_OD): Compound (+)-**1**.

Figure 7 ^1^H NMR (600 MHz, CD_3_OD): Compound **2**.

Figure 8 ^13^C NMR (150 MHz, CD_3_OD): Compound **2**.

Figure 9 ^1^H–^1^H-COSY (CD_3_OD): Compound **2**.

Figure 10 ^1^H–^13^C HSQC (CD_3_OD): Compound **2**.

Figure 11 ^1^H–^13^C HMBC (CD_3_OD): Compound **2**.

Figure 12 ^1^H–^1^H-NOESY (CD_3_OD): Compound **2**.

Figure 13 ^1^H NMR (600 MHz, CD_3_OD): Compound (+)-**3**.

Figure 14 ^13^C NMR (150 MHz, CD_3_OD): Compound (+)-**3**.

Figure 15 ^1^H–^1^H-COSY (CD_3_OD): Compound (+)-**3**.

Figure 16 ^1^H–^13^C-HSQC (CD_3_OD): Compound (+)-**3**.

Figure 17 ^1^H–^13^C-HMBC (CD_3_OD): Compound (+)-**3**.

Figure 18 ^1^H–^1^H-NOESY (CD_3_OD): Compound (+)-**3**.

1. **Computational Details**

For each compound (+)-**1** and (+)-**3**, a conformational analysis was performed using Spartan ’10^[^[^1^](#_ENREF_1)^]^ at a semiempirical level of theory (PM6).^[^[^2^](#_ENREF_2)^]^ Geometry optimizations and frequency calculations at DFT level as well as the calculation of electronic excitations using time-dependent DFT^[^[^3^](#_ENREF_3)^]^ were performed using Gaussian 16, Rev. C.01.^[^[^4^](#_ENREF_4)^]^ Geometry optimization of each conformer was performed with the B3LYP functional,^[^[^5-8^](#_ENREF_5)^]^ the Pople basis set 6-311+G(2d,p)^[^[^9-11^](#_ENREF_9)^]^ and the IEFPCM solvation model^[^[^12^](#_ENREF_12)^]^ for methanol. For the simulation of electronic CD spectra, the electronic excitations were calculated based on the DFT optimized conformers. Based on the calculated values of each conformer, Boltzmann averaged CD spectra were created using SpecDis v1.71.^[^[^13^](#_ENREF_13)^]^ An empirical shift (in the range of -30–30 nm) and an empirical bandwidth (0.1–0.3 eV) were applied for the comparison of simulated with measured data.

The respective xyz coordinate files of the geometry optimized conformers can be found in a separate ZIP file.

**Input Lines**

**Conformer Distribution**

SEARCHMETHOD=THOROUGH FINDBOATS KEEPALL CONF_SELECTION_RULE=5

**Geometry Optimization**

#p opt=tight freq b3lyp 6-311+G(2d,p) scrf=(iefpcm,solvent=methanol)

**Electronic Excitations**

#p td=(nstates=60) b3lyp 6-311+G(2d,p) scrf=(iefpcm,solvent=methanol)

1. **ECD Spectra**


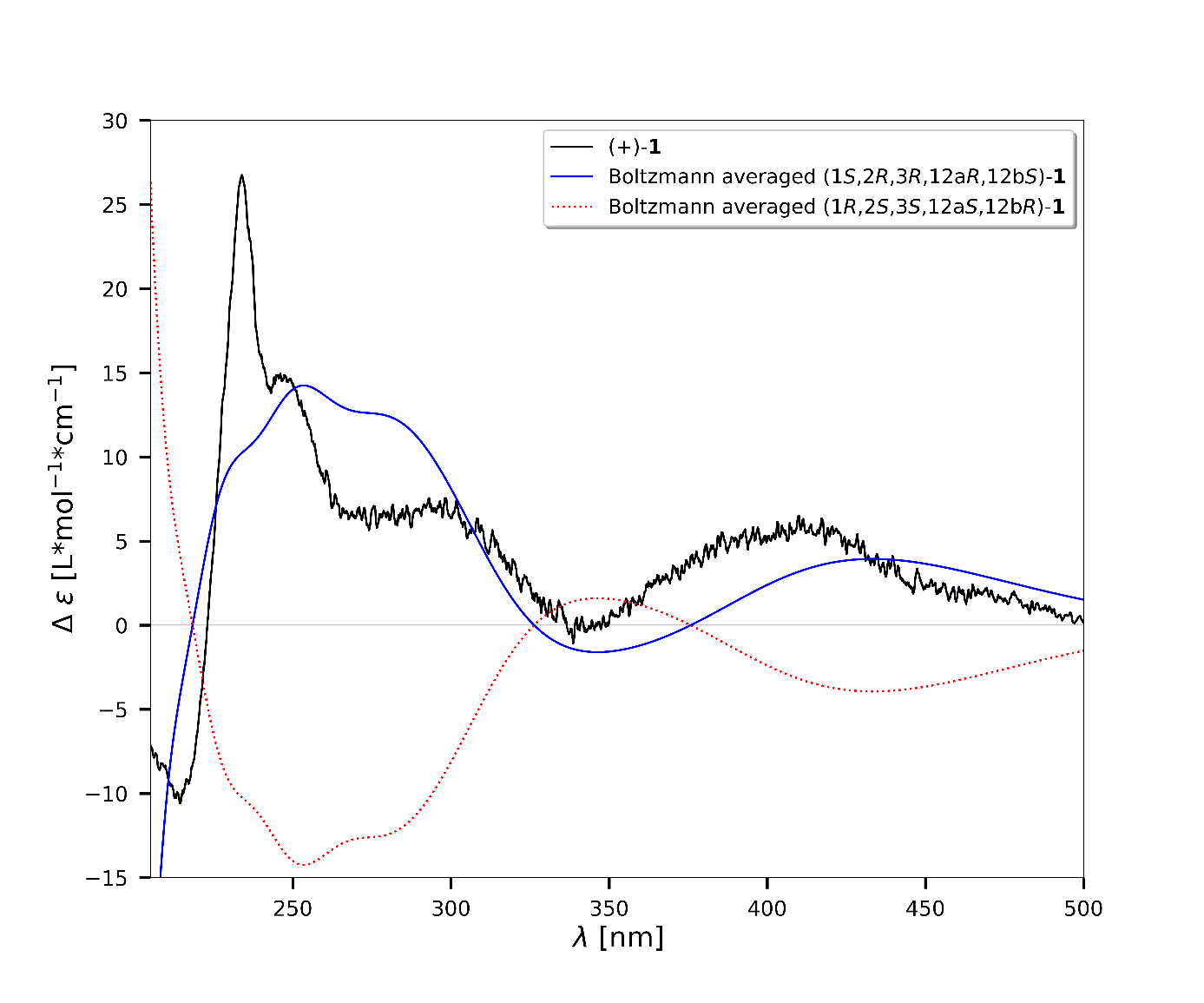


| Similarity factor (1*S*,2*R*,3*R*,12a*R*,12b*S*) = | 0.843 |
| --- | --- |
| Similarity factor (1*R*,2*S*,3*S*,12a*S*,12b*R*) = | 0.009 |
| Δ (Enantiomeric Similarity Index) = | 83.4% |

Figure 19 Comparison of the observed (+)-**1** (c = 0.1 mM in MeOH; black) with the Boltzmann averaged calculated CD spectra (1*S*,2*R*,3*R*,12a*R*,12b*S*; 205–500 nm; -8 nm shift; 0.3 eV band width; blue). Similarity factors for both possible enantiomers as well as the resulting Δ(ESI), determined with SpecDis v1.71.^[^[^13-15^](#_ENREF_13)^]^


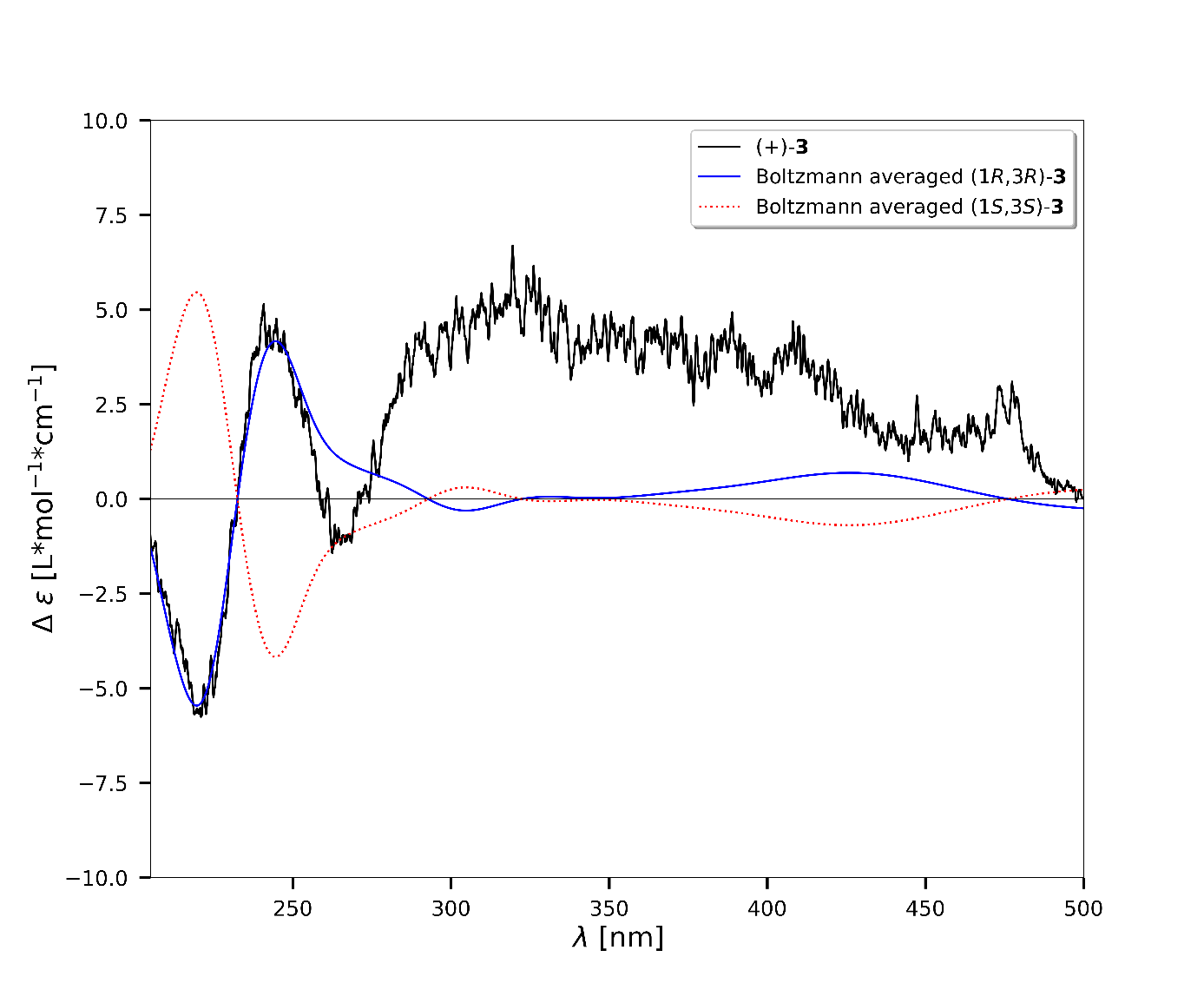


| Similarity factor (1*R*,3*R*) = | 0.542 |
| --- | --- |
| Similarity factor (1*S*,3*S*) = | 0.025 |
| Δ (Enantiomeric Similarity Index) = | 51.7% |

Figure 20 Comparison of the observed (+)-**3** (c = 0.1 mM in MeOH; black) with the Boltzmann averaged calculated CD spectra (1*R*,3*R*; 205–500 nm; +21 nm shift; 0.3 eV band width; blue). Similarity factors for both possible enantiomers as well as the resulting Δ(ESI), determined with SpecDis v1.71.^[^[^13-15^](#_ENREF_13)^]^

1. **HPLC Data**

Figure 21 UV-Spectrum of compound (+)-**1**

Figure 22 Chromatogram at 300 nm of compound (+)-**1**. Measured with Lichrospher 100 RP18 column (125 x 4 mm, 5 µm, Macherey-Nagel) using water and acetonitrile; Flow 1 mL/min. Starting with 99% water and 1% acetonitrile, to 100 % acetonitrile in 20 minutes. 5 minutes at 100 % acetonitrile.

Figure 23 UV-Spectrum of ATX-I

Figure 24 Chromatogram at 300 nm of ATX-I. Measured with Lichrospher 100 RP18 column (125 x 4 mm, 5 µm, Macherey-Nagel) using water and acetonitrile; Flow 1 mL/min. Starting with 99% water and 1% acetonitrile, to 100 % acetonitrile in 20 minutes. 5 minutes at 100 % acetonitrile.

Figure 25 Chromatogram at 300 nm of compound (+)-**3**. Measured with Lichrospher 100 RP18 column (125 x 4 mm, 5 µm, Macherey-Nagel) using water and acetonitrile; Flow 1 mL/min. Starting with 99% water and 1% acetonitrile, to 100 % acetonitrile in 20 minutes. 5 minutes at 100 % acetonitrile.

Figure 26 UV-Spectrum of compound (+)-**3**

Figure 27 Chromatogram at 300 nm of compound (+)-**2**. Measured with Lichrospher 100 RP18 column (125 x 4 mm, 5 µm, Macherey-Nagel) using water and acetonitrile; Flow 1 mL/min. Starting with 99% water and 1% acetonitrile, to 100 % acetonitrile in 20 minutes. 5 minutes at 100 % acetonitrile.

Figure 28 UV-Spectrum of compound (+)-**2**

1. **HPLC/MS Data**


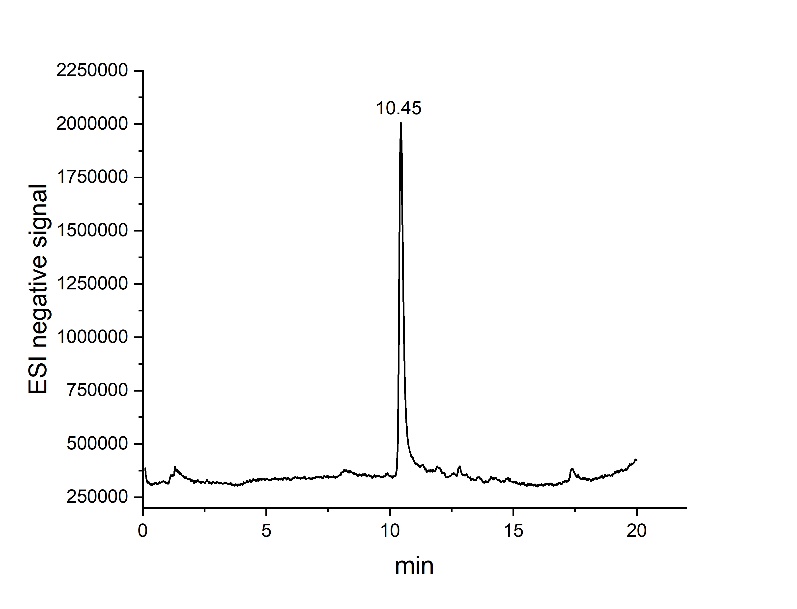


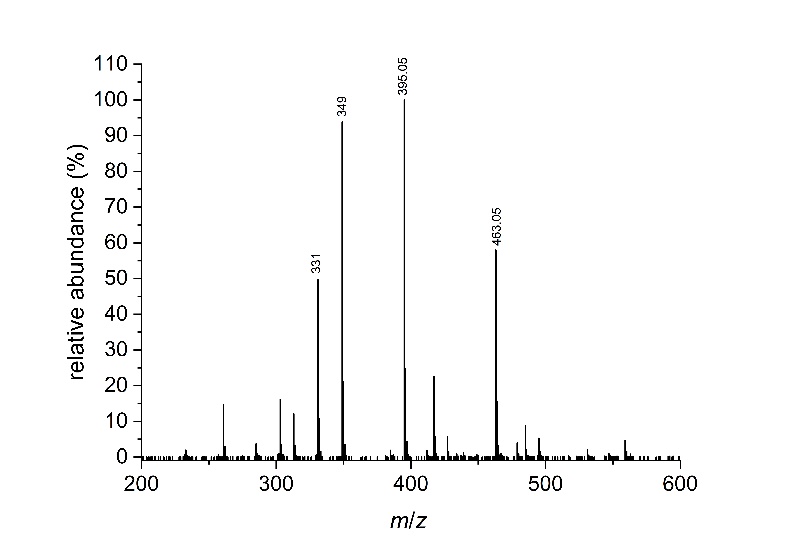
ATX-I

(+)-**1**

**
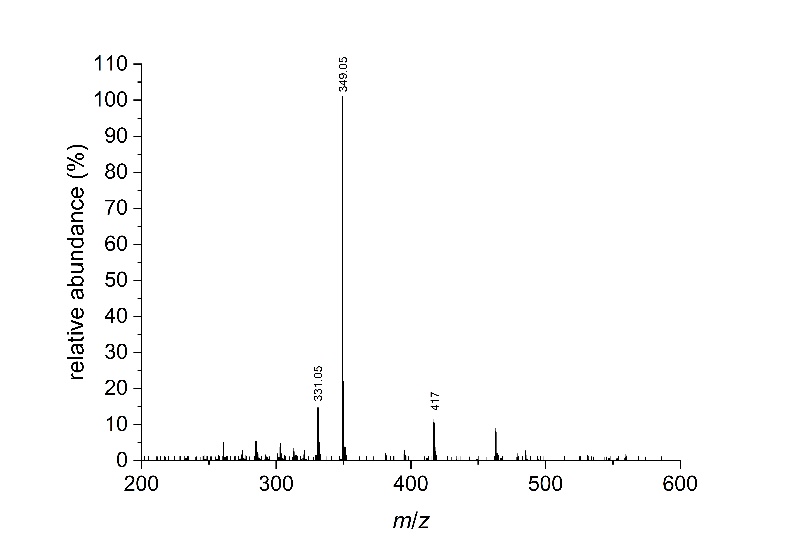
**
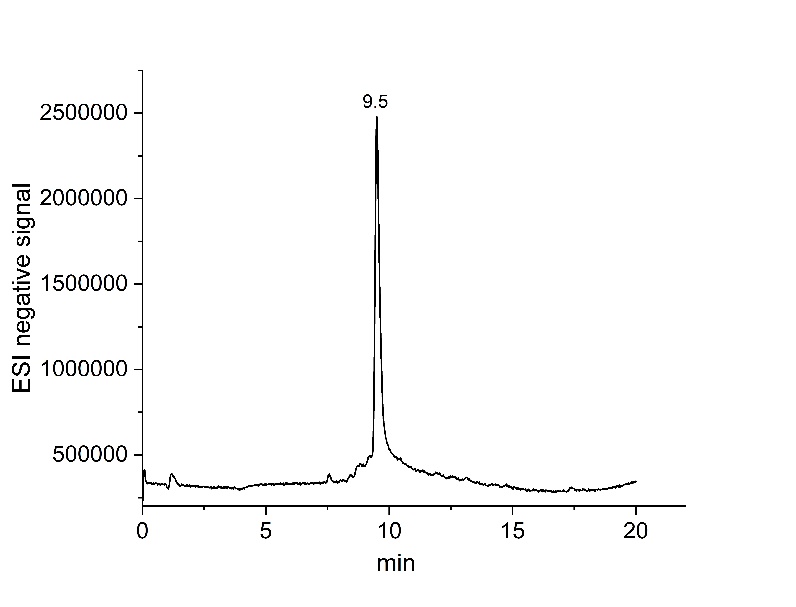


(+)-**2**


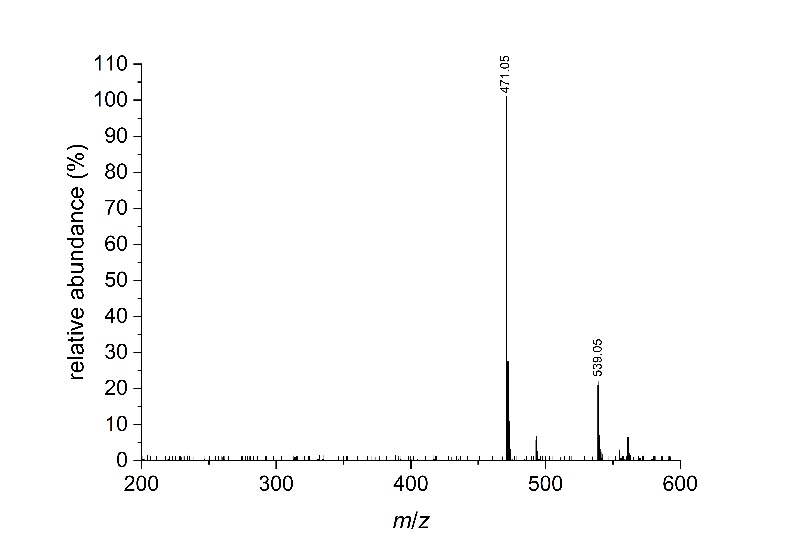

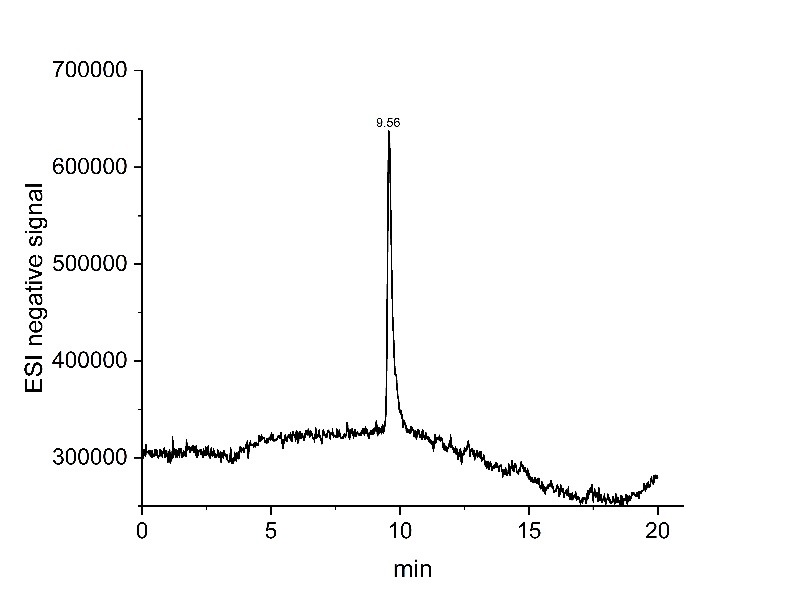


(+)-**3**


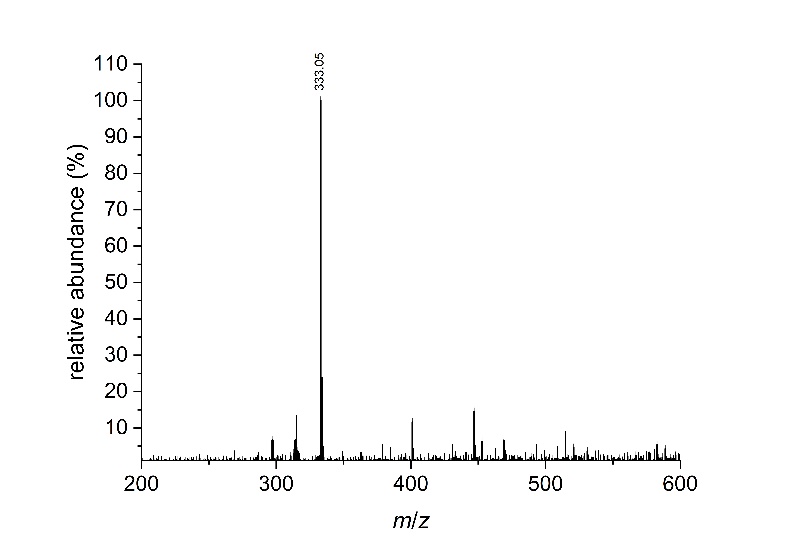


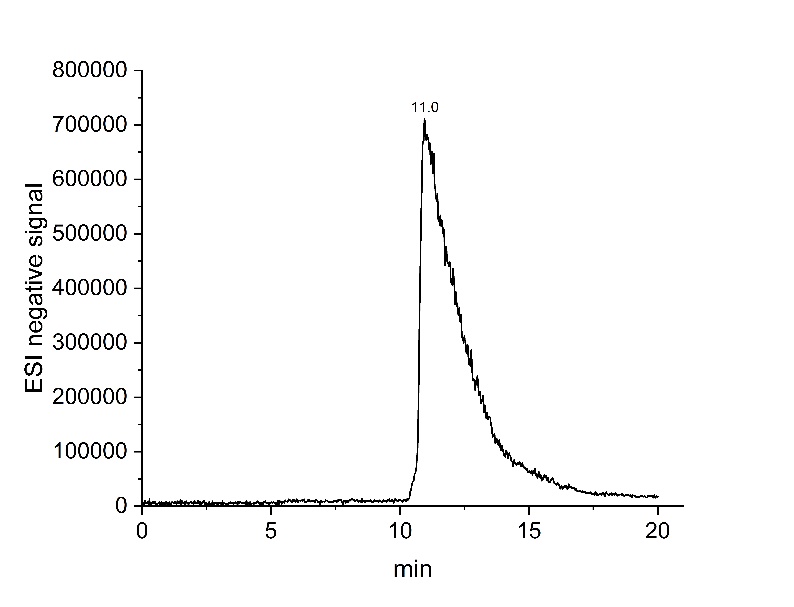


Figure 29 Mass spectra of ATX-I and compounds **1**-**3** obtained using an ESI interface. Analyses of the purified compounds by detection of ESI-negative signal fragments and single-quadrupole mass spectra showing the quasi-molecular ions.

The mass UV spectra were analyzed with a Shimadzu HPLC-LCMS-2020 mass spectrometer ﬁtted with a Superspher RP18 column (125 x 2 mm, 4 µm particle size, Merck). The chromatographic conditions consisted of a gradient from 1% to 100% acetonitrile in 20 min, and an isocratic step at 100% acetonitrile for 1 min at 40^0^C and 10 µl injection volume was used. The ﬂow rate was 0.45 ml/min. The detector voltage was set to 1.1 kV, the interface voltage was set to -3.5 kV in the negative ESI mode, DL-Voltage was set to 1.5 V, Q-Array RF-Voltage was set to 60 V and the evaporator temperature was set to 400°C.


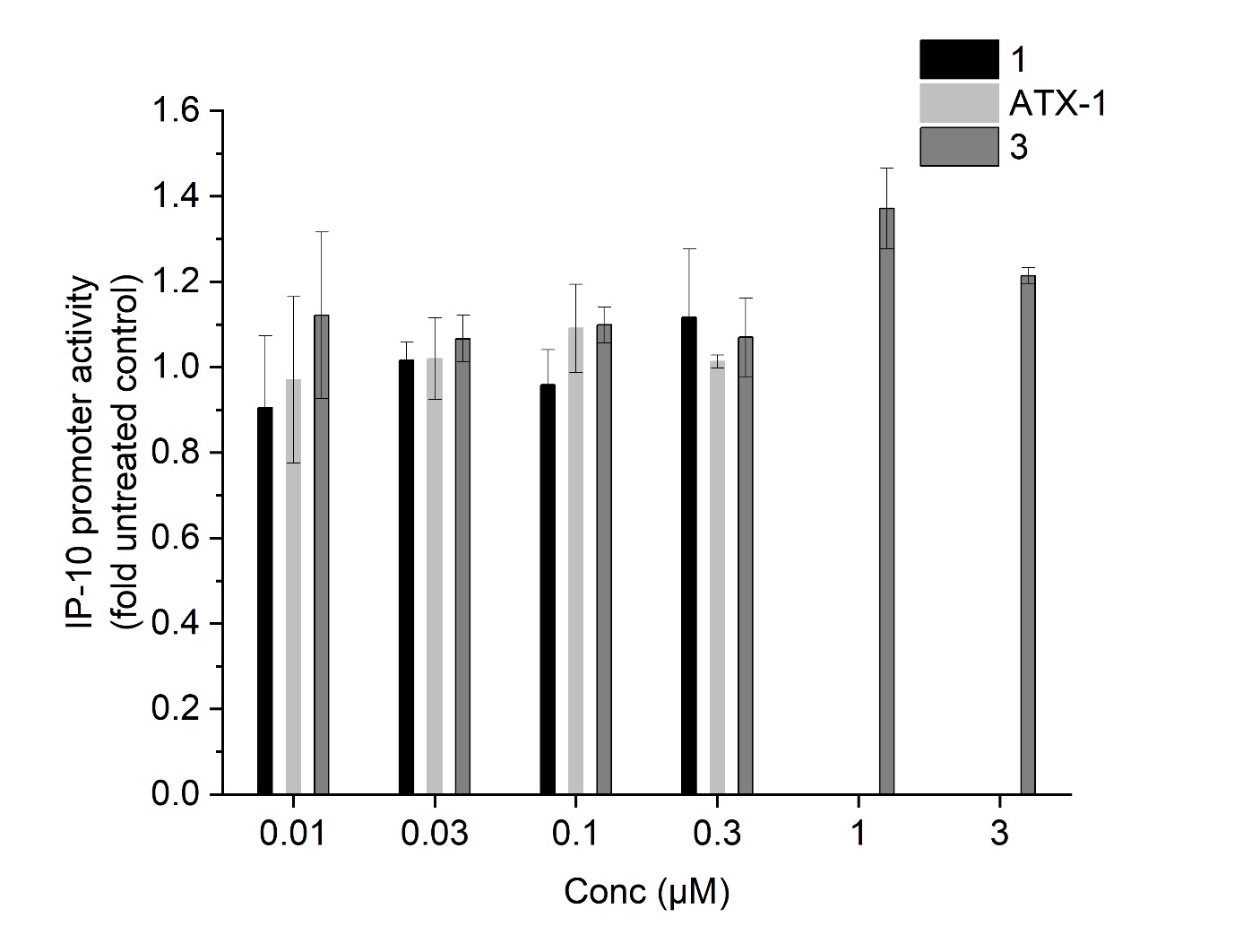


Figure 30 Influence of compounds (+)-**1**, (+)-**3**, and ATX-1 on IP-10 (CXCL-10) promoter activity.

BEAS-2B cells were transiently transfected with a human IP-10-promoter dependent reporter plasmid by electroporation and stimulated with 10 ng/mL TNF-α, 10 ng/mL IFN-γ and 5 ng/mL IL-1β for 24 h with and without test compounds. Control: stimulation only. The expression of the reporter gene was determined as described in the experimental section. Data represent the mean ± SEM of at least three independent experiments.

Sequencing results of the ITS1-5.8S rDNA-ITS2 region of nuclear DNA of the producer strain with ITS4 und ITS5 primer.^[^[^16^](#_ENREF_16)^]^

Sequenz forward (5‘🡪3‘):

ATTACACAATATGAAGGCGGGCTGGACACCCTCCAGCCGGGCACTGCTTCACGGCGTGCGCGGCTGGAGCCGGCCCTGCTGAATTATTCACCCGTGTCTTTTGCGTACTTCTTGTTTCCTGGGTGGGCTCGCCCGCCATCAGGACCAACCATAAACCTTTTTGTAATAGCAATCAGCGTCAGTAACAACGTAATTAATTACAACTTTCAACAACGGATCTCTTGGTTCTGGCATCGATGAAGAACGCAGCGAAATGCGATACGTAGTGTGAATTGCAGAATTCAGTGAATCATCGAATCTTTGAACGCACATTGCGCCCTTTGGTATTCCAAAGGGCATGCCTGTTCGAGCGTCATTTGTACCCTCAAGCTTTGCTTGGTGTTGGGCGTCTTTTGTCTCCAGCTCGCTGGAGACTCGCCTTAAAGTCATTGGCAGCCGGCCTACTGGTTTCGGAGCGCAGCACAAGTCGCGCTCTTGCCCAGCCAAGGTCAGCGTCCAGCAAGCCTTTTTTCAACCTTTGACCTCGGATCAGGTAGGGATACCCGCTGAACTTAAGCATATCAA

Sequenz reverse (5‘🡪3‘):

AAAGGTTGAAAAAAGGCTTGCTGGACGCTGACCTTGGCTGGGCAAGAGCGCGACTTGTGCTGCGCTCCGAAACCAGTAGGCCGGCTGCCAATGACTTTAAGGCGAGTCTCCAGCGAGCTGGAGACAAAAGACGCCCAACACCAAGCAAAGCTTGAGGGTACAAATGACGCTCGAACAGGCATGCCCTTTGGAATACCAAAGGGCGCAATGTGCGTTCAAAGATTCGATGATTCACTGAATTCTGCAATTCACACTACGTATCGCATTTCGCTGCGTTCTTCATCGATGCCAGAACCAAGAGATCCGTTGTTGAAAGTTGTAATTAATTACGTTGTTACTGACGCTGATTGCTATTACAAAAAGGTTTATGGTTGGTCCTGATGGCGGGCGAGCCCACCCAGGAAACAAGAAGTACGCAAAAGACACGGGTGAATAATTCAGCAGGGCCGGCTCCAGCCGCGCACGCCGTGAAGCAGTGCCCGGCTGGAGGGTGTCCAGCCCGCCTTCATATTGTGTAATGATCCCTCCGCAGGTTCACCTACGGAGACCTTGTTACGC

1. **References**

[1] Wavefunction, Inc., Irvine, CA, USA, **2009**.

[2] J. J. P. Stewart, *J. Mol. Model.* **2007**, *13*, 1173-1213.

[3] E. Runge, E. K. U. Gross, *Physical Review Letters* **1984**, *52*, 997-1000.

[4] M. J. Frisch, G. W. Trucks, H. B. Schlegel, G. E. Scuseria, M. A. Robb, J. R. Cheeseman, G. Scalmani, V. Barone, G. A. Petersson, H. Nakatsuji, X. Li, M. Caricato, A. V. Marenich, J. Bloino, B. G. Janesko, R. Gomperts, B. Mennucci, H. P. Hratchian, J. V. Ortiz, A. F. Izmaylov, J. L. Sonnenberg, Williams, F. Ding, F. Lipparini, F. Egidi, J. Goings, B. Peng, A. Petrone, T. Henderson, D. Ranasinghe, V. G. Zakrzewski, J. Gao, N. Rega, G. Zheng, W. Liang, M. Hada, M. Ehara, K. Toyota, R. Fukuda, J. Hasegawa, M. Ishida, T. Nakajima, Y. Honda, O. Kitao, H. Nakai, T. Vreven, K. Throssell, J. A. Montgomery Jr., J. E. Peralta, F. Ogliaro, M. J. Bearpark, J. J. Heyd, E. N. Brothers, K. N. Kudin, V. N. Staroverov, T. A. Keith, R. Kobayashi, J. Normand, K. Raghavachari, A. P. Rendell, J. C. Burant, S. S. Iyengar, J. Tomasi, M. Cossi, J. M. Millam, M. Klene, C. Adamo, R. Cammi, J. W. Ochterski, R. L. Martin, K. Morokuma, O. Farkas, J. B. Foresman, D. J. Fox, Gaussian 16, Revision C.01, Wallingford, CT, **2019**.

[5] S. H. Vosko, L. Wilk, M. Nusair, *Can. J. Phys.* **1980**, *58*, 1200-1211.

[6] C. Lee, W. Yang, R. G. Parr, *Phys. Rev. B* **1988**, *37*, 785-789.

[7] A. D. Becke, *Int. J. Chem. Phys.* **1993**, *98*, 5648-5652.

[8] A. D. Becke, *Phys. Rev. A* **1988**, *38*, 3098.

[9] M. J. Frisch, J. A. Pople, J. S. Binkley, *Int. J. Chem. Phys.* **1984**, *80*, 3265-3269.

[10] R. Krishnan, J. S. Binkley, R. Seeger, J. A. Pople, *Int. J. Chem. Phys.* **1980**, *72*, 650-654.

[11] T. Clark, J. Chandrasekhar, G. W. Spitznagel, P. V. R. Schleyer, *J. Comput. Chem.* **1983**, *4*, 294-301.

[12] J. Tomasi, B. Mennucci, E. Cancès, *Comput. Theor. Chem* **1999**, *464*, 211-226.

[13] T. Bruhn, A. Schaumlöffel, Y. Hemberger, G. Pescitelli, Berlin, Germany, **2017**.

[14] T. Bruhn, A. Schaumlöffel, Y. Hemberger, G. Bringmann, *Chirality* **2013**, *25*, 243-249.

[15] E. Debie, E. De Gussem, R. K. Dukor, W. Herrebout, L. A. Nafie, P. Bultinck, *ChemPhysChem* **2011**, *12*, 1542-1549.

[16] T. J. White, T. Bruns, S. Lee, J. Taylor, in *PCR Protocols* (Eds.: M. A. Innis, D. H. Gelfand, J. J. Sninsky, T. J. White), Academic Press, San Diego, **1990**, pp. 315-322.
